# LncRNA *U90926* is dispensable for the development of obesity‐associated phenotypes in vivo

**DOI:** 10.14814/phy2.15901

**Published:** 2024-01-03

**Authors:** Bristy Sabikunnahar, Sydney Caldwell, Stella Varnum, Tyler Hogan, Karolyn G. Lahue, Birgit Rathkolb, Raffaele Gerlini, Nathalia R. V. Dragano, Antonio Aguilar‐Pimentel, Martin Irmler, Adrián Sanz‐Moreno, Patricia da Silva‐Buttkus, German Mouse Clinic Consortium, Oana V. Amarie, Lore Becker, Dirk H. Busch, Julia Calzada‐Wack, Yi‐Li Cho, Lillian Garrett, Sabine M. Hoelter, Markus‐Josef Kraiger, Stefanie Leuchtenberger, Manuela A. Östereicher, Nadine Spielmann, Claudia Stoeger, Wolfgang Wurst, Johannes Beckers, Eckhard Wolf, Valerie Gailus‐Durner, Helmut Fuchs, Martin Hrabe de Angelis, Jennifer L. Ather, Matthew E. Poynter, Dimitry N. Krementsov

**Affiliations:** ^1^ Department of Biomedical and Health Sciences University of Vermont Burlington Vermont USA; ^2^ Institute of Experimental Genetics and German Mouse Clinic Helmholtz Zentrum München Neuherberg Germany; ^3^ German Center for Diabetes Research (DZD) Neuherberg Germany; ^4^ Institute of Molecular Animal Breeding and Biotechnology, Gene Center Ludwig‐Maximilians‐University München Munich Germany; ^5^ TUM School of Life Sciences Technische Universität München Freising Germany; ^6^ Department of Medicine University of Vermont Burlington Vermont USA

**Keywords:** adipogenesis, LncRNA, obesity, U90926

## Abstract

Obesity is a global health problem characterized by excessive fat accumulation, driven by adipogenesis and lipid accumulation. Long non‐coding RNAs (lncRNAs) have recently been implicated in regulating adipogenesis and adipose tissue function. Mouse lncRNA *U90926* was previously identified as a repressor of in vitro adipogenesis in 3T3‐L1 preadipocytes. Consequently, we hypothesized that, in vivo, *U90926* may repress adipogenesis, and hence its deletion would increase weight gain and adiposity. We tested the hypothesis by applying *U90926*‐deficient (U9‐KO) mice to a high‐throughput phenotyping pipeline. Compared with WT, U9‐KO mice showed no major differences across a wide range of behavioral, neurological, and other physiological parameters. In mice fed a standard diet, we have found no differences in obesity‐related phenotypes, including weight gain, fat mass, and plasma concentrations of glucose, insulin, triglycerides, and free fatty acids, in U9‐KO mice compared to WT. *U90926* deficiency lacked a major effect on white adipose tissue morphology and gene expression profile. Furthermore, in mice fed a high‐fat diet, we found increased expression of *U90926* in adipose tissue stromal vascular cell fraction, yet observed no effect of *U90926* deficiency on weight gain, fat mass, adipogenesis marker expression, and immune cell infiltration into the adipose tissue. These data suggest that the *U90926* lacks an essential role in obesity‐related phenotypes and adipose tissue biology in vivo.

## 1 INTRODUCTION

Obesity is characterized by the abnormal and excessive accumulation of adipose tissue, primarily white adipose tissue (WAT), that is harmful to human health. Increasingly prevalent worldwide, obesity is a major risk factor for type 2 diabetes, nonalcoholic fatty liver disease, cardiovascular disease, and several types of cancer (Beuther & Sutherland, 2007; Haslam & James, 2005). Expansion of adipose tissue and obesity occur when adipocyte number or size increases (Jo et al., 2009; Tang & Lane, 2012). Generally, preadipocytes differentiate into mature adipocytes by adipogenesis to increase adipocyte number, which has a profound effect on obesity (Ali et al., 2013). Understanding molecular mechanisms and modulation of adipogenesis is essential to combat obesity.

Obesity is often described as a chronic, low‐grade systemic inflammation characterized by increased pro‐inflammatory cytokines produced by adipocytes and the infiltration of leukocytes, mainly macrophages, into adipose tissue (Rouault et al., 2013; Wernstedt Asterholm et al., 2014). Adipose tissue macrophages (ATMs) play a vital role in obesity by regulating adipocyte function, decreasing efferocytosis, and secreting pro‐inflammatory cytokines and chemokines that directly affect inflammation and metabolic imbalance, for example, insulin resistance (Boutens & Stienstra, 2016; da Costa et al., 2016). The interplay between ATMs and adipocytes during obesity is regulated by many molecular factors, which is an ongoing area of research interest (Cheng et al., 2019; Weisberg et al., 2003).

Similar to the other areas of cell biology, long non‐coding RNAs (lncRNAs) have emerged as essential regulators in adipocyte development (Sun et al., 2013). LncRNAs are unique transcripts that are 200 nucleotides in size, often polyadenylated, and usually do not encode proteins (Derrien et al., 2012). They possess crucial regulatory effects in many biological processes, such as transcriptional repression, X chromosome inactivation, chromatin remodeling, cell differentiation, and cancer metastasis (Clemson et al., 1996; Engreitz et al., 2016; Gupta et al., 2010; Lin et al., 2016; Wang et al., 2014). Recently, studies have shown that several lncRNAs, including *SRA*, *lnc‐RAPn*, *slincRAD*, *PU.1 AS*, *HOTAIRiADINR*, and *NEAT1*, regulate white preadipocyte differentiation (Divoux et al., 2014; Gernapudi et al., 2015; Pang et al., 2013; Xiao et al., 2015; Yi et al., 2013).

We have recently characterized a murine lncRNA named *U90926*. We found that it is highly expressed in macrophages upon toll‐like receptor (TLR) activation and is protective during endotoxic shock in mice (Krementsov et al., 2014; Sabikunnahar et al., 2023). Other groups have characterized *U90926* as a co‐factor of herpes simplex visus (HSV)‐1 replication and a modulator of neutrophil migration during ischemic brain injury (Chen et al., 2021; Shirahama et al., 2020). Prior to these studies, Chen et al. (2017) described the first function of *U90926*: a repressor of adipogenesis (Chen et al., 2017). First, Chen et al. (2017) showed a downregulation of *U90926* expression in WAT in *ob/ob* and *db/db* mice, as well as mice fed a high‐fat diet (HFD). Using 3T3‐L1 preadipocytes as an in vitro model of adipogenesis, they demonstrated that lncRNA *U90926* was constitutively expressed in preadipocytes, and its expression decreased dramatically as they differentiated into mature adipocytes. Functionally, knockdown of *U90926* expression promoted adipogenesis in 3T3‐L1 cells, and *U90926* overexpression repressed it. Based on this study, we hypothesized that, in vivo, deletion of *U90926* would promote adipogenesis and, therefore, weight gain and adiposity. To test our hypothesis, we employed a *U90926* knockout mouse model (U9‐KO) that we recently generated, which carries a deletion of all five exons of the *U90926* gene (Sabikunnahar et al., 2023). Here, we have evaluated different parameters of obesity‐related phenotypes using both a regular diet and a high‐fat diet to determine the effect of *U90926* on adiposity. We also used a high‐throughput phenotyping pipeline to assess any additional physiological effects of *U90926* deficiency. Both approaches found minimal effects of *U90926* deficiency, suggesting that unlike its role in inflammation, this gene is not essential for adipose tissue function and other physiological traits.

## 2 MATERIALS AND METHODS

### 2.1 Animals

*U90926*‐deficient mice (U9‐KO) lacking all five exons of the *U90926* gene on the C57BL/6J background were generated previously, and PCR confirmation of the U9‐KO genotype was performed as previously described (Sabikunnahar et al., 2023). For the German Mouse Clinic (GMC, Munich, Germany) phenotyping experiments, littermate control WT C57BL/6J and homozygous U9‐KO mice were generated from U9‐KO heterozygous breeders (het x het crosses) to minimize any cage/parental effects. The animals were shipped to GMC at 7 weeks of age and subjected to the standard screening pipeline starting at 9 weeks of age (Fuchs et al., 2011, 2018). The phenotyping cohort was comprised of 14 U9‐KO and 11 WT female mice, and 11 U9‐KO and 14 WT male mice. Diet‐induced obesity experiments were performed at the University of Vermont animal facility, as follows. Eight‐week‐old WT and U9‐KO mice were switched from a normal diet (ND) to a high‐fat diet (HFD, 60% kcal fat) purchased from Research Diets Inc. (catalog #D12492). For these experiments, WT control C57BL/6J (B6) mice were initially generated from U9‐KO het x het crosses, then bred as homozygous x homozygous U9‐KO or WT for 2–4 generations to generate homozygous experimental animals. Mice were maintained on 12‐h light–dark cycles. Food was provided ad libitum and replaced weekly.

### 2.2 Cell culture

3T3‐L1‐MBX mouse embryonic fibroblasts were purchased from American Type Culture Collection (RRID: CVCL_0A20, Manassas, VA) and cultured and differentiated according to manufacturer recommendations. For adipogenesis assays, 3T3‐L1 preadipocytes were grown to confluence in DMEM with 10% FBS (Gibco); after 2 days, the culture medium was replaced with differentiation medium containing 10% FBS, 1 μM dexamethasone (Sigma), 0.5 mM 3‐isobutyl‐1‐methylxanthie (IBMX) (Sigma), and 1 μg/mL insulin (Sigma). Two days later, the medium was changed to maintenance media (DMEM with 10% FBS + 1 μg/mL insulin). and maintained until Day 8, with media changes every 2 days. At each time point of the experiment, cells were collected in TRIzol reagent (Invitrogen, Waltham, MA) for RNA extraction. Bone marrow‐derived macrophages (BMDMs) were isolated, cultured, and stimulated with LPS, as described previously (Sabikunnahar et al., 2023).

### 2.3 RNA extraction and quantitative reverse transcription PCR (RT‐qPCR)

For both in vitro and in vivo experiments, Direct‐zol RNA Microprep Kits (Zymo Research, Irvine, CA) were used for RNA extraction as per the manufacturer's protocol. Fat tissue samples were collected into 2 mL screw‐cap tubes containing 1/3 full of 1.0 mm diameter Silicon‐Carbide particles (Biospec Products, Bartlesville, OK) and 1 mL of TRIzol reagent (Invitrogen, Waltham, MA) per tube. Then, the samples were homogenized using a Mini‐Beadbeater instrument by Biospec Products (Bartlesville, OK) for a total of 2 min by 4 cycles, and tubes were placed in an ice‐water bath during each interval to avoid overheating. Then, tubes were centrifuged at 13,000 rpm for 2 min to pellet any debris, 200 μL of homogenate was collected, and 100 μL of fresh TRIzol was added, followed by extraction using the Direct‐zol RNA Microprep Kit as above.

RNA concentration was determined by Nanodrop (Thermo Scientific NanoDrop 2000 Spectrophotometer), and cDNA synthesis reaction by reverse transcription was performed according to the manufacturer's instructions using the qScript cDNA Super MIX kit (QuantBio, Beverly, MA). The qPCR reaction was performed according to the manufacturer's instructions using the DyNAmo ColorFlash SYBR Green kit, and target‐specific primers were ordered from Thermo Fisher Scientific (Waltham, MA). All the primer sequences were adapted from Chen et al. (2017) except the *B2m* primers (forward: 5′‐CATGGCTCGCTCGGTGACC‐3′ and reverse: 5′‐AATGTGAGGCGGGTGGAACTG‐3′). The qPCR was run on a Quant Studio 3 Real‐Time PCR System by Applied Biosystems (Thermo Fisher Scientific, Waltham, MA). Target gene expression was normalized by the expression of the housekeeping genes (HKG), either 18S rRNA or *B2m* (β‐2‐Microglobulin), as indicated, and calculated by a comparative Ct method formula of 2^−∆∆Ct^ and multiplied by a factor of 10,000 for ease of visualization, that is, $2^{-{\mathrm{Ct}}_{\text{target}}-{\mathrm{Ct}}_{\mathrm{HKG}}}\times 10,000$. We did not normalize relative expression to a reference sample (i.e., delta‐deltaCt).

### 2.4 Fat content analysis by DEXA and NMR

Fat content measurements were performed at three different time points using both dual‐energy x‐ray absorptiometry (DEXA) and nuclear magnetic resonance (NMR) techniques following the GMC pipeline. DEXA was carried out consecutively using a Faxitron Ultrafocus equipped with a 10 × 15 cm CMOS detector (Faxitron Bioptics, LLC, Tucson, AZ, USA). The mouse was anesthetized, and prior to imaging weight and length were recorded. Subsequently, the animal was fixed on an x‐ray‐permeable plate and placed in the instrument. Image acquisition and analysis were performed using VisionDXA software (Faxitron Bioptics, LLC). Quantitative DEXA measures were computed automatically for the whole mouse. Two mice (WT = 1 male and U9‐KO = 1 male) were excluded from the DEXA analysis because of the failure in automatic skull segmentation performed by the DXA software. The whole‐body composition was measured using nuclear magnetic resonance imaging (Bruker MiniSpec LF 50), a non‐invasive and reliable method for measuring lean tissue and body fat in live mice without anesthesia. The mice were placed in the NMR instrument, and the measurements were obtained following the manufacturer's protocol.

### 2.5 Plasma analysis

As part of the metabolic screening at GMC, fasting plasma glucose and other compounds were measured from mice subjected to food withdrawal overnight, and blood was collected. The screen was performed using a Beckman‐Coulter AU 480 autoanalyzer and adapted reagents from Beckman‐Coulter (Krefeld, Germany), except that free fatty acids (NEFA) were measured using a kit from Wako Chemicals GmbH (NEFA‐HR2, Wako Chemicals, Neuss, Germany). In the primary screen, a broad set of biomarkers was measured, including various enzyme activities, as well as plasma concentrations of specific substrates and electrolytes in ad libitum‐fed mice. Plasma insulin levels or insulin and leptin were determined using an electroluminescence‐linked immunosorbent assay from Mesoscale Discovery (mouse/rat insulin kit K152BZC; mouse metabolic kit K15124C) and a MESO QuickPlex SQ 120 Sector imager (Mesoscale Discovery, Rockville, Maryland, USA).

### 2.6 Intraperitoneal glucose tolerance test (IpGTT)

At GMC, mice were used for the glucose tolerance test after a 6–7‐h‐long food withdrawal. Before food withdrawal and at the beginning of the test, the body weight of mice was determined. For the determination of the fasting blood glucose level, the tip of the tail was scored using sterilized scissors, and a small drop of blood was analyzed with the Accu‐Chek Aviva glucose analyzer (Roche/Mannheim). Thereafter, mice were injected intraperitoneally with 2 g of glucose/kg body weight using a 20% glucose solution, a 25‐gauge needle, and a 1‐mL syringe. 15, 30, 60, and 120 min after glucose injection, additional blood samples (one drop each) were collected and used to determine blood glucose levels as described before. Repeated bleeding was induced by removing the clot from the first incision and massaging the tail of the mouse.

### 2.7 Histopathological and molecular analysis (RNA‐seq) of perigonadal white adipose tissue

At GMC, mice of both sexes were euthanized by CO_2_ inhalation at the age of 16 weeks. After macroscopic observations of the internal organs, including subcutaneous (sWAT) and visceral/perigonadal white adipose tissue (pWAT) depots, the pWAT pads were weighed. One portion of pWAT was snap frozen and used for molecular analysis, while the other was fixed in formalin and embedded in paraffin. Sections (4 μm) of pWAT were stained with hematoxylin and eosin (H&E) for histological analysis. In addition, 28 tissues were also collected and histologically analyzed according to previously described standardized protocols (Fuchs et al., 2018). Two independent pathologists performed the histopathological evaluation.

For molecular phenotyping, the pWAT was collected from the mice and analyzed by RNA‐seq. First, total RNA was isolated employing the RNeasy Mini kit (Qiagen), including Trizol treatment. The Agilent 2100 Bioanalyzer was used to assess RNA quality, and RNA with RIN >7 was used for RNA‐seq analysis. RNA‐seq was performed on an Illumina NovaSeq 6000 with a PE150‐stranded protocol. Paired‐end data was generated and analyzed by an RNA‐seq pipeline consisting of quality control (FastQC, MultiQC), read trimming (trim_galore), genome alignment (STAR), and gene‐level read counting (summarizeOverlaps, mode = “Union”). Significantly differentially expressed genes were determined using the DEseq2 tool (Love et al., 2014) in R studio after filtering low‐expressed genes (<10 reads). Statistical analysis was performed using *t*‐tests with Benjamini‐Hochberg multiple testing correction (Padj). Detection *p*‐values were used to exclude background signals; significant genes were filtered for detection *p* < 0.05 in more than half of the samples in at least one group per comparison. To identify significant differentially expressed genes, we used a standard cutoff of Padj <0.05 without a fold change filter. RNA‐seq data has been submitted to the GEO database at NCBI (GSE240645).

### 2.8 Flow cytometry

Perigonadal visceral WAT was collected, the stromal vascular cell (SVC) fraction was separated, and the mature adipocyte fraction was enriched using a collagenase‐based approach, as described previously (Cho et al., 2014). Red blood cell (RBC) lysis was performed on SVC by incubating the cells in a 0.8% ammonium chloride solution (StemCell Technologies, Cambridge, MA), and the cells were subjected to staining for flow cytometry analysis. Cells were stained with the UV LIVE/DEAD fixable stain (Invitrogen) and then surface labeled for different combinations of the following markers: CD45, CD11b, CD11c, CD206, CD301, CD19, TCRβ, Ly6G, F4/80, and Ly6C (Biolegend, San Diego, CA) and fixed with 1% paraformaldehyde (Sigma Aldrich, St. Louis, MO). Samples were analyzed on an LSRII cytometer (BD Biosciences) or an Aurora cytometer (Cytek Biosciences). All flow cytometry data analysis was performed using FlowJo Software version 10.6.1 (BD Biosciences).

### 2.9 Statistical analyses

Statistical analyses not pertaining to RNA‐seq data were carried out using GraphPad Prism software version 9.2.0 (GraphPad Software, San Diego, CA, USA). The specific tests used to assess the significance of the observed differences are detailed in the figure legends. All center values represent the mean, and error bars represent the standard error of the mean. The significance of differences was determined by a two‐way ANOVA, with Šidák post hoc test performed for pairwise comparison between the indicated groups throughout the paper, unless otherwise specified. A *p*‐value of <0.05 was considered significant.

## 3 RESULTS

### 3.1 LncRNA U90926 is expressed in pWAT and 3T3‐L1 preadipocytes

**FIGURE 1:**
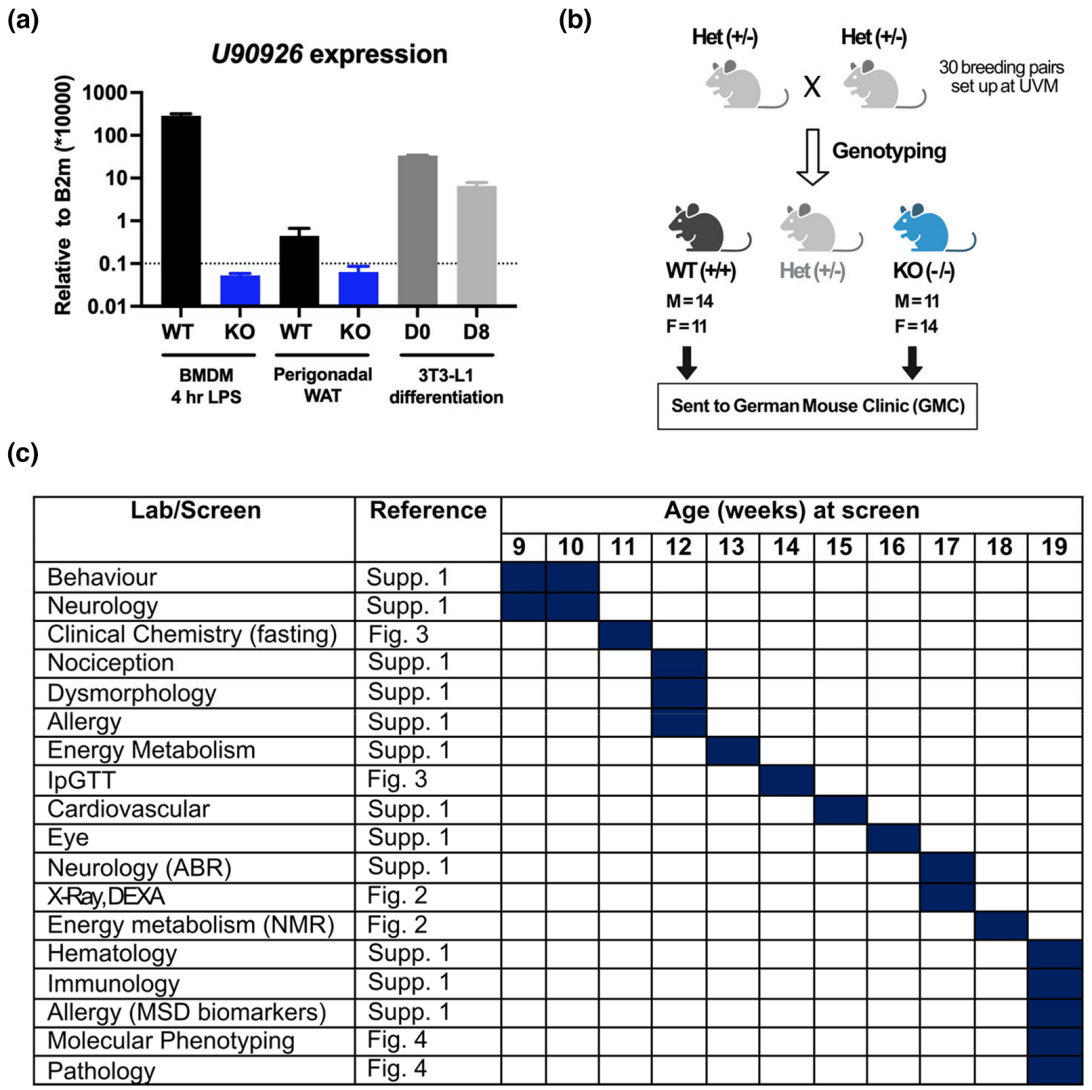
Long non‐coding RNA *U90926* expression in perigonadal white adipose tissue (pWAT) and phenotyping screening of U9‐KO mice. (a) Bone marrow‐derived macrophages (BMDMs) and pWAT were isolated from WT B6 and U9‐KO mice. BMDMs were stimulated with LPS (100 ng/mL) for 4 h. 3T3‐L1 cells were collected at two time points of differentiation, days D0 (Day 0) and D8 (Day 8). All the samples were subjected to RT‐qPCR for *U90926* expression measurement. All the data are expressed relative to the housekeeping gene *B2m* and calculated by the comparative Ct method formula 2^−∆∆Ct^ and multiplied by a factor of 10,000 for ease of visualization. The data are represented as the mean ± SEM. A Student's *t‐*test was performed for each sample type. (b) Schematic representation of the WT and U9‐KO mice that were sent to the German Mouse Clinic (GMC) for phenotyping screening. (c) Table showing the lab or screening that was performed on the WT and U9‐KO mice from 9 to 19 weeks of age at GMC and the location of the data in this manuscript.

Previous studies have documented a role for *U90926* as a repressor of adipogenesis in vitro and its downregulation in the fat depots of obese mice (Chen et al., 2017). This led us to investigate the effect of the *U90926* deletion on weight gain and obesity‐related phenotypes in mice. We have previously generated a U9‐KO mouse model on the C57BL/6J (B6) background, which we utilized here to test this hypothesis in vivo. First, we validated the *U90926* expression in our models. We found that *U90926* is expressed at detectable levels in pWAT depots from WT B6 mice, with an 85% reduction in expression in U9‐KO mice as measured by RTq‐PCR to levels below the limit of reliable detection (Figure 1a). We also compared the *U90926* expression level in WAT with LPS‐stimulated bone marrow‐derived macrophages (BMDMs), which we previously determined to exhibit very high levels of *U90926* expression, and with 3T3‐L1 cells before and after differentiation. We found that *U90926* expression in WT WAT, while detectable, was orders of magnitude lower compared with LPS‐stimulated BMDMs and 3T3‐L1 preadipocytes, with the latter downregulating *U90926* expression after differentiation into mature adipocytes (Figure 1a), as previously reported (Chen et al., 2017). Taken together, we have shown that *U90926* is expressed in pWAT and cultured adipocytes, and we were able to reproduce the previously reported findings that *U90926* expression declines as the cells approach differentiation. The apparent low level of expression of *U90926* in whole WAT is likely due to its low expression in mature adipocytes.

### 3.2 High‐throughput physiological phenotyping of U90926‐deficient mice

**FIGURE 2:**
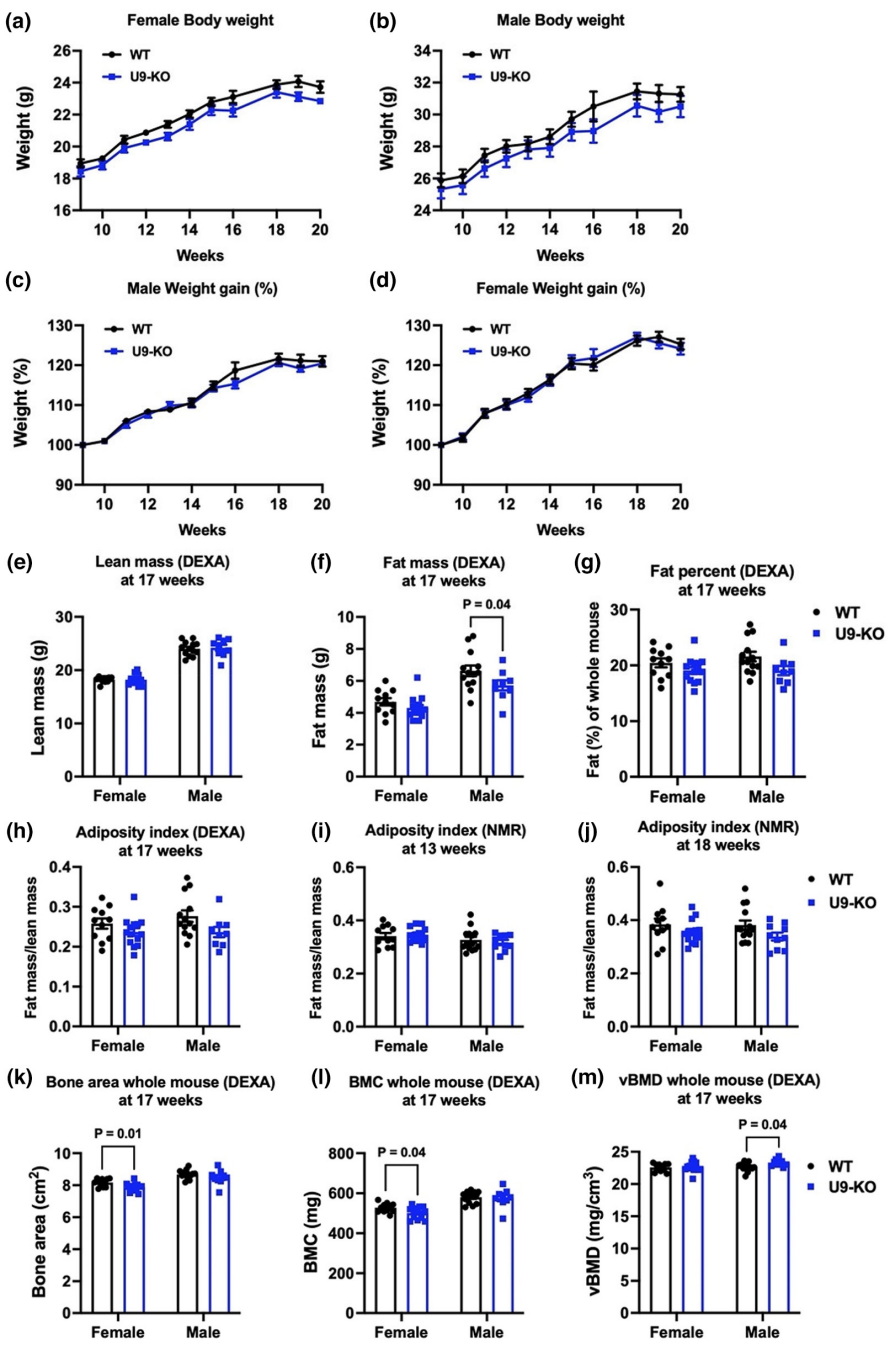
WT and U9‐KO mice show comparable total body weight and fat content. (a–d) are showing the whole‐body weight of female (WT, *n* = 11; U9‐KO, *n* = 14) and male (WT, *n* = 14; U9‐KO, *n* = 11) mice, respectively, to compare the difference between WT and U9‐KO mice. (e–h) At 17 weeks, the same mouse groups as above were analyzed by dual‐energy x‐ray absorptiometry (DEXA) for whole‐body lean and fat content and adiposity index. (i) and (j) show two time points at 13 weeks and 18 weeks, respectively, at which the WT and U9‐KO mice mentioned above were analyzed by NMR to compare their adiposity index. The adiposity index was calculated as the fat mass/lean mass of a mouse. (k–m) The 17‐week‐old mice mentioned above were also analyzed by DEXA for whole mouse bone area, bone mineral content (BMC), and volumetric bone mineral density (vBMD). The data are represented as the mean ± SEM. A two‐way ANOVA was used to assess the significance of differences between WT and U9‐KO mice. For multiple comparisons, Fisher's LSD test was used in panels (e–g), whereas for other panels, our standard Šídák test was performed.

**FIGURE 3:**
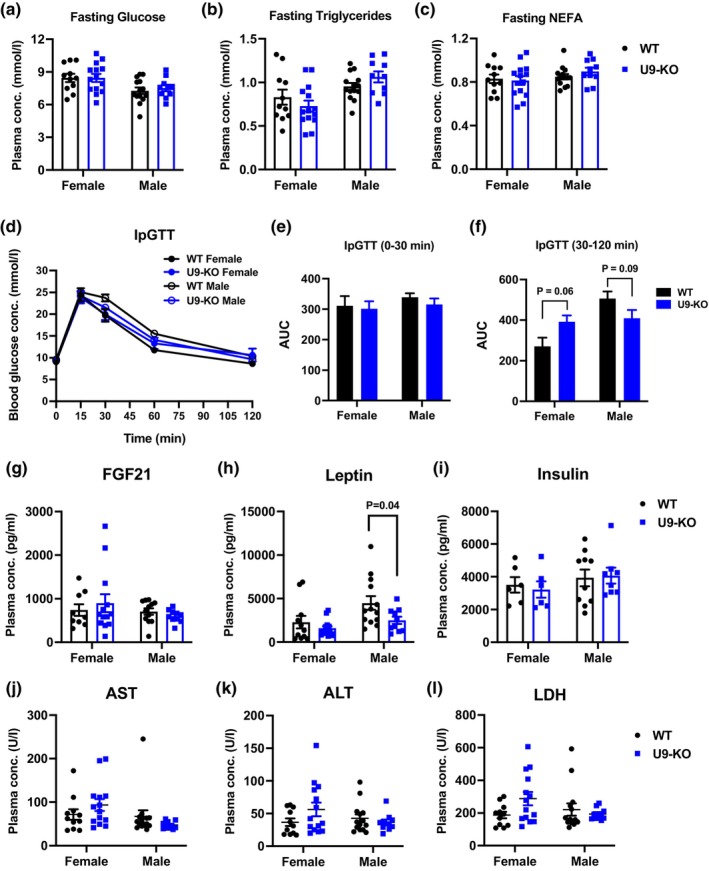
*U90926* deficiency has no major metabolic effect in vivo. (a–c) Blood was collected from WT and U9‐KO mice after the overnight withdrawal of food. Then, plasma concentrations of glucose, triglycerides, and non‐esterified fatty acids (NEFA) were measured. (d–f) Intraperitoneal glucose tolerance test (IpGTT) was performed on WT and U9‐KO mice fasting for 6–7 h, where glucose concentration was measured at the beginning, *T* = 0 and at 15, 30, 60, and 120 min after glucose administration in panel (d). Data show area under the curve (AUC) analysis for 0–30 min in panel (e), and for 30–120 min in panel (f). (g–i) Blood was collected from 13‐week‐old WT and U9‐KO mice to measure the plasma concentration of FGF21 (g), leptin (h), and insulin (i) by an electroluminescence‐linked immunosorbent assay based on the Mesoscale Discovery (MSD) technology. (j–l) Blood was collected from ad libitum‐fed WT and U9‐KO mice to measure the plasma concentration of liver enzymes, aspartate transaminase (AST), alanine aminotransferase (ALT), and lactate dehydrogenase (LDH), as depicted in panels (j–l), respectively. Mice groups used for all the above experiments are 13‐week‐old WT (*n* = 14 males *n* = 11 females) and U9‐KO (*n* = 10 males *n* = 14 females). A two‐way ANOVA was performed for statistical analysis to assess the significance of differences between WT and U9‐KO mice.

**FIGURE 4:**
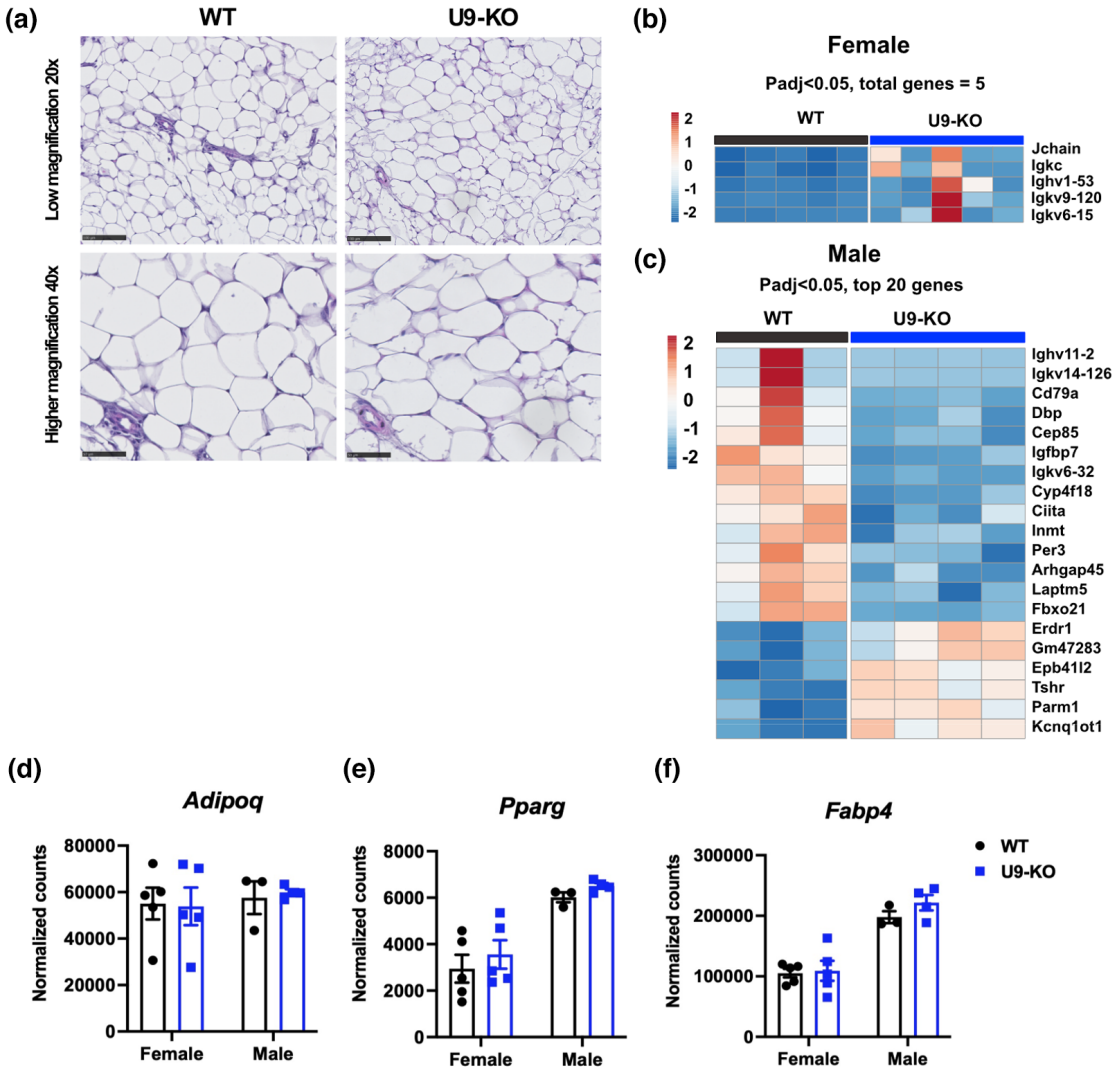
Histological and transcriptional analysis shows no major obesity‐related phenotypes in white adipose tissue (WAT) from WT and U9‐KO mice. (a) Histological aspect of the perigonadal WAT H&E stained. The images are representative of WT (*n* = 5) (left) and U9KO mice (*n* = 5) (right), respectively. Note that the adipocytes in both groups are similar in shape and size and show the typical morphology of containing one large cytoplasmic lipid droplet (unilocular), displacing the nucleus to the periphery. (b–f) RNA‐seq analysis was performed on RNA isolated from whole perigonadal adipose tissue collected from WT and U9‐KO mice. DEseq2 analysis was done to identify the differentially expressed genes in U9‐KO versus WT tissues. Heatmaps show the topmost differentially expressed genes based on the Padj < 0.05 cutoff value in females (b) and males (c). Adipogenesis markers, *Adipoq*, *Pparγ*, and *Fabp4* counts in RNA‐seq data were compared in WT and U9‐KO mice in panels (d–f), respectively.

We next performed a detailed phenotypic characterization of U9‐KO mice on a ND in collaboration with the German Mouse Clinic (GMC, Munich, Germany), using their standard mouse phenotyping pipeline (see Materials and Methods). We generated a cohort of control C57BL/6J WT (*M* = 14, *F* = 11) and homozygous U9‐KO (*M* = 11, *F* = 14) mice at the University of Vermont for the GMC pipeline, which were littermate offspring from U9‐KO heterozygous breeders, thus minimizing any cage or littermate effects (Figure 1b). Phenotyping details, the timing of experimentation, and the location of results are shown in Figure 1c. Generally, U9‐KO mice developed normally and exhibited minimal effects on all of the tested physiological parameters, including morphology, behavior, neurology, and hematology (Data S1), with the exception of several bone mineral parameters, as described below. Phenotyping results specifically related to adipose tissue homeostasis are provided in Figures 2, 3, 4 and described in detail in the following sections.

### 3.3 Deletion of U90926 has no effect on weight gain and fat mass in vivo

First, to determine if *U90926* grossly affects weight gain in mice fed a normal diet, body weight was measured weekly from 9 to 20 weeks of age. We found no significant difference in weight gain in female and male U9‐KO mice compared to WT (Figure 2a–d). Since excessive fat accumulation is the major hallmark of obesity, we determined whether *U90926* deficiency impacts the total fat mass. We measured whole mouse fat mass and lean mass by two different methods, DEXA and time domain nuclear magnetic resonance (NMR), at different time points (weeks 13, 17, and 18). We found no significant differences in overall lean mass and fat mass accumulation in U9‐KO compared to WT (Figure 2e,f; Data S1), with the exception of fat mass measured by DEXA analysis in male mice at 17 weeks, which was decreased in U9‐KO (Figure 2f). However, this difference was no longer significant when fat accumulation was calculated as a percent of the whole mouse (Figure 2g). The adiposity index, calculated as the ratio of fat mass to lean mass, showed no significant differences in either male or female U9‐KO and WT mice (Figure 2h–j). Since bone density can also contribute to total body mass, we also examined the bone mineral parameters of the WT and U9‐KO mice by DEXA at 17 weeks of age. DEXA analysis showed that bone area of the whole mouse and bone mineral content (BMC) were decreased in U9‐KO females compared with WT females (Figure 2k,l), respectively. However, volumetric bone mineral density (vBMD), which is calculated as the ratio of the BMC divided by the bone area, was slightly increased in male U9‐KO mice compared with WT males (Figure 2m). Taken together, these findings suggest that loss of *U90926* does not affect overall weight gain or total fat mass accumulation under a normal diet, but it may exert some subtle sex‐specific effects on bone homeostasis.

### 3.4 Obesity‐related metabolic changes are unaltered in U9‐KO mice

Obesity is a significant risk factor for numerous metabolic disorders, such as type 2 diabetes and nonalcoholic fatty liver disease, which are accompanied by insulin resistance and elevated liver enzymes, respectively (Liu et al., 2021). In obesity, metabolic consequences leading to insulin resistance include an increased concentration of glucose, lipids, and free fatty acids in circulation (Kahn & Flier, 2000). Here, we investigated plasma concentrations of glucose, triglycerides (TG), and non‐esterified fatty acids (NEFA) in WT and U9‐KO mice after overnight food withdrawal. We found that *U90926* deficiency does not affect fasting glucose, TG, or NEFA concentrations (Figure 3a–c). To gain a deeper understanding of glucose homeostasis and how obesity affects metabolism, we conducted intraperitoneal glucose tolerance tests (IpGTT). We found no difference in glucose clearance between WT and U9‐KO mice, indicating that U9‐KO mice do not have impaired glucose tolerance (Figure 3d–f). Our metabolic screening also showed that *U90926* deficiency does not impact other major plasma biomarkers of obesity, such as fibroblast growth factor 21 (FGF21), leptin, and insulin (Figure 3g–i). Although there was a subtle decrease in leptin concentration in the male U9‐KO mice compared with the WT (*p* = 0.042), their body weight, fat mass, food intake, and other metabolic parameters do not suggest altered leptin signaling.

Obesity is associated with many liver diseases, such as nonalcoholic fatty liver disease (NAFLD), nonalcoholic steatohepatitis (NASH), and cryptogenic cirrhosis (Marchesini et al., 2008). Like other liver injuries, these obesity‐associated liver diseases are also accompanied by elevated liver enzymes such as aspartate aminotransferase (AST) and alanine aminotransferase (ALT) in the circulation (Marchesini et al., 2008). Given that, we measured the plasma concentrations of AST, ALT, and lactate dehydrogenase (LDH) enzymes, and found no significant differences in the levels of liver enzymes in U9‐KO mice compared to WT (Figure 3j–l). Similarly, no histological differences were detected in liver, pancreas, or interscapular brown adipose tissue comparing the WT and U9‐KO mice (data not shown). Altogether, these data demonstrate that the *U90926* deletion does not affect glucose homeostasis and other obesity‐related metabolic parameters in vivo.

### 3.5 U90926 deficiency does not impact pWAT histological and transcriptional signatures

Since adipocyte size and the number increase in pWAT during obesity (Jo et al., 2009), we examined the effect of the *U90926* deletion on WAT morphology in vivo. We detected no apparent differences in adipocyte size and shape when comparing control WT with U9‐KO mice (Figure 4a). Both groups showed the typical morphology of WAT‐adipocytes, containing one large cytoplasmic lipid droplet that takes up almost the entire cell and leads to the displacement of the nucleus to the periphery. No differences in vascularization, the presence of lymphoid cells, and/or fibrosis between the groups were observed.

Adipogenesis involves a sequential transcriptional cascade that controls adipocyte development at the molecular level (Ahmad et al., 2020). Knockdown of *U90926* in differentiating 3T3‐L1 preadipocytes has been reported to induce the upregulation of genes encoding adipogenesis markers, including peroxisome proliferator‐activated receptor gamma (PPARγ), CCAAT/enhancer‐binding protein α (C/EBPα), fatty acid binding protein 4 (FABP4), and adiponectin (AdipoQ) (Chen et al., 2017). Based on this, we hypothesized that the *U90926* deletion in vivo may affect molecular signatures related to adipogenesis and obesity, even in the absence of overt histological or metabolic changes. We performed RNA‐seq analysis on pWAT depots collected from WT and U9‐KO mice. Differentially expressed genes (DEGs) between WT and KO tissues were identified using the DEseq2 package (see Materials and Methods). Using a standard cutoff of Padj <0.05, 36, and 5 DEGs were found in males and females, respectively, with no overlapping DEGs between the sexes and zero significant DEGs found when males and females were analyzed together (Figure 4b,c). The DEGs showed relatively heterogenous expression across samples and contained no obvious adipogenesis markers or other obesity‐related molecular markers. Direct examination of the mRNA‐encoding adipogenesis markers AdipoQ, PPARg, and FABP4 in the RNA‐seq data also showed no difference in U9‐KO compared with WT (Figure 4d–f), altogether suggesting that *U90926* deficiency in vivo has minimal effects on obesity‐related gene expression in pWAT. Taken together with the histologic assessment and adiposity index measurements, these data suggest that the *U90926* deficiency in vivo does not affect the major characteristics of WAT.

### 3.6 Diet‐induced obesity phenotypes are not different in U9‐KO mice

**FIGURE 5:**
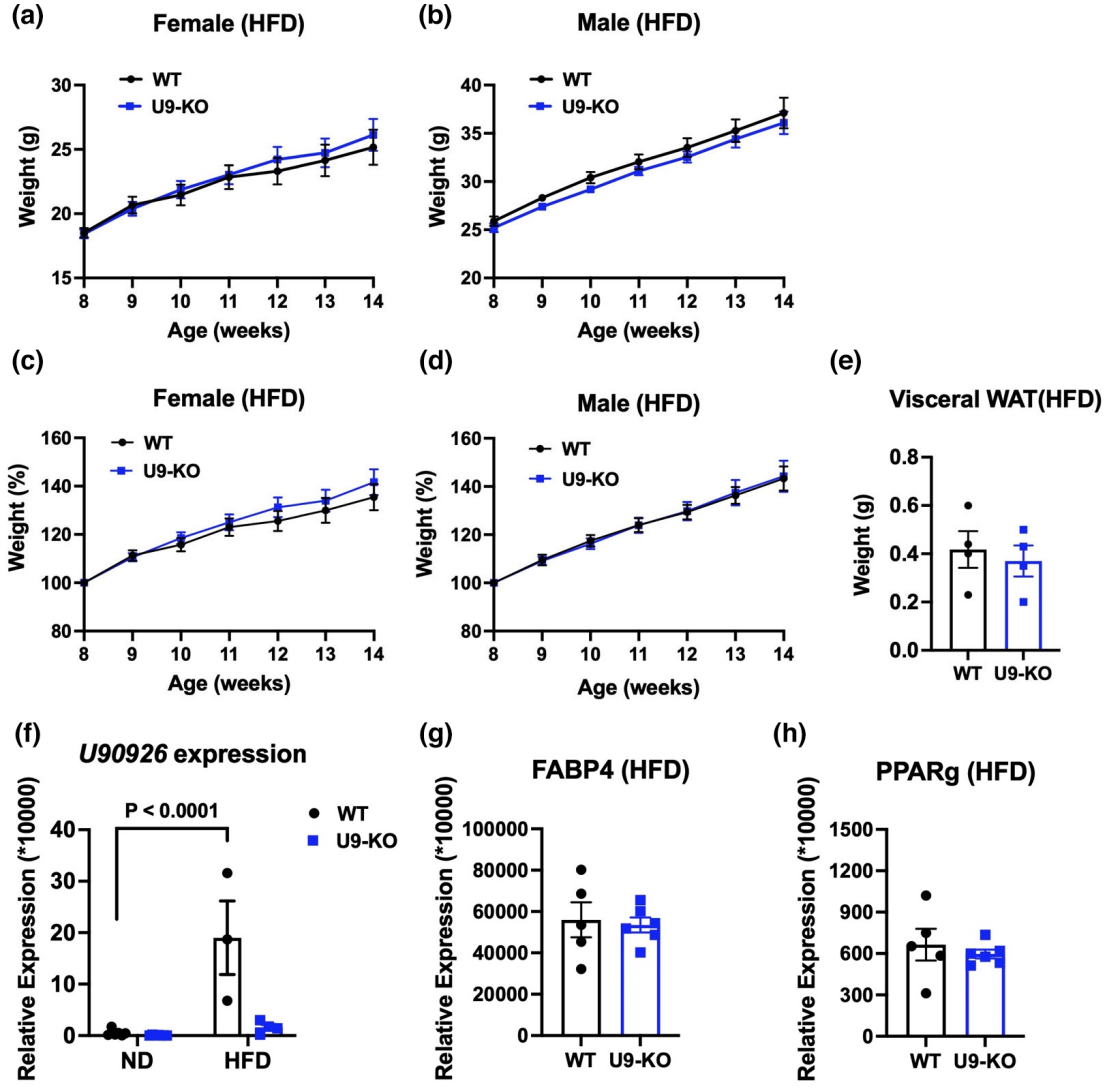
*U90926* deletion does not alter obesity phenotypes during diet‐induced obesity. (a–d) WT and U9‐KO mice were exposed to a high‐fat diet (HFD) containing 60% fat at 8 weeks of their age, and body weight was measured every week for 6 weeks. Weight gain in gram (g) and percentage (%) of female (WT = 10, U9‐KO = 11) and male (WT = 7, U9‐KO = 12) mice upon HFD are shown here in panels (a–d), respectively. (e) Visceral/perigonadal fat depots were collected from female HFD‐fed WT and U9‐KO mice (*n* = 4 in each group) for comparison in WAT weight. (f) *U90926* expression in perigonadal WAT was measured by RT‐qPCR in tissues from normal diet (ND)‐ and HFD‐fed WT and U9‐KO mice. The ND mice used here were age‐matched and generated in parallel experiments with HFD mice using the same homozygous x homozygous breeding strategy. (g, h) Adipogenesis markers, such as *Fabp4* and *Pparg*, were measured by RT‐qPCR in perigonadal WAT collected from HFD‐fed WT and U9‐KO mice. All RT‐qPCR data are expressed relative to the housekeeping gene Beta‐2‐microglobulin (B2m) in panel (f) and 18SrRNA in panels (g, h) and calculated by a comparative Ct method formula of 2^−∆∆Ct^ and multiplied by a factor of 10,000 for ease of visualization. The data are represented as the mean ± SEM. The significance of differences between the indicated groups was determined by a two‐way ANOVA (a–d, f) and a Student's *t*‐test (e, g, h).

In the studies above, we demonstrated a lack of effect of the *U90926* deletion on spontaneous weight gain or adiposity in vivo in mice fed a normal diet (ND). Next, to test whether *U90926* deficiency influences pathologic weight gain during obesity, we used a standard diet‐induced obesity model (Wang & Liao, 2012), in which we utilized a HFD containing 60% kcal of fat. To confirm the robustness of this model, we first compared weight gain in WT mice fed a HFD to those fed a ND from 8 to 16 weeks of age. As expected, HFD‐fed WT mice of both sexes exhibited much greater weight gain compared with sex‐matched ND‐fed WT mice (Figure S3). Next, we introduced the HFD to control WT mice and U9‐KO mice at the age of 8 weeks and continued for an additional 6 weeks. We found no difference in weight gain between WT and U9‐KO mice fed a HFD (Figure 5a–d). Perigonadal fat weights were also comparable between WT and U9‐KO mice (Figure 5e). These data indicate that *U90926* does not exert a major effect on weight gain and fat accumulation during diet‐induced obesity.

A previous study showed that HFD‐induced obese mice have decreased *U90926* expression in their subcutaneous and visceral WAT compared to ND‐fed mice, with expression of genes encoding adipogenesis markers PPARγ, FABP4, and AdipoQ upregulated in WAT of HFD‐induced obese mice and in U9‐knockdown 3T3‐L1 preadipocytes (Chen et al., 2017). In contrast, our results demonstrated that WT HFD obese mice have significantly higher *U90926* expression in the pWAT than do ND‐fed mice (Figure 5f), with no differences in *Fabp4* and *Pparg* expression in visceral WAT of HFD‐fed WT and U9‐KO mice (Figure 5g,h).

**FIGURE 6:**
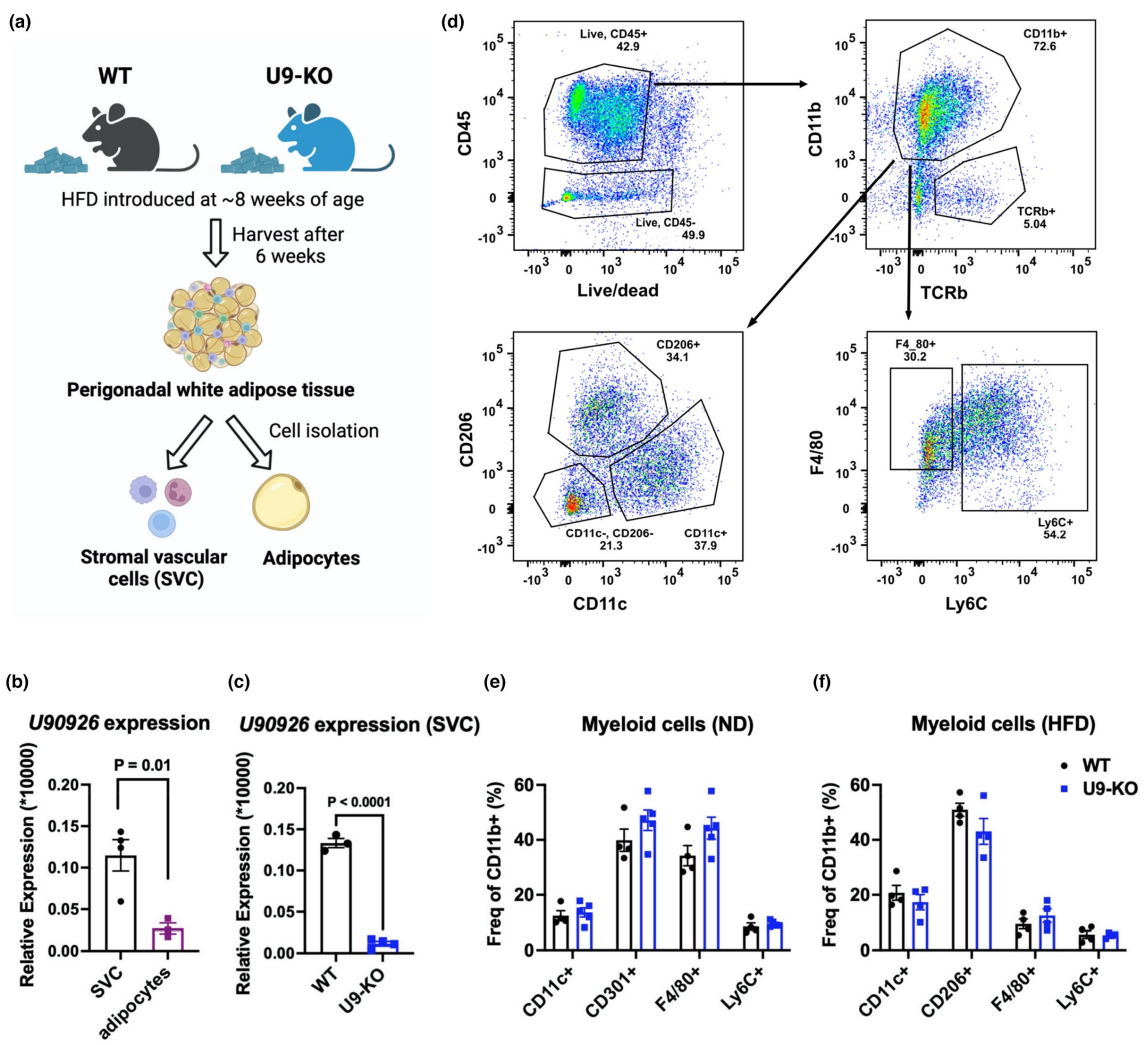
Lack of *U90926* does not alter immune cell infiltration in the perigonadal adipose tissue. (a) Schematic diagram of stromal vascular cell (SVC) content and mature adipocyte isolation from perigonadal white adipose tissue (pWAT). (b) pWAT from high‐fat diet (HFD)‐fed WT mice was collected, and SVC and mature adipocytes were separated, followed by RT‐qPCR to measure *U90926* expression in each cellular fraction. (c) SVC was collected from HFD‐fed WT and U9‐KO mice, and RT‐qPCR was performed to measure *U90926* expression. The data (b, c) are expressed relative to the housekeeping gene 18SrRNA and calculated by the comparative Ct method formula 2^−∆∆Ct^ and multiplied by a factor of 10,000 for ease of visualization. Data are represented as the mean ± SEM, and a Student's *t*‐test was performed for comparison. (d) Gating scheme of flow cytometry analysis of SVC (e, f) isolated from pWAT. Here, leukocytes (CD45^+^) were gated, followed by gating for myeloid cells (CD11b^+^) and T cells (TCRb^+^). The CD11b^+^ TCRb‐myeloid population was used as the parent gate for calculating the frequencies of all the myeloid cell sub‐populations. (e, f) SVCs were collected from ND‐fed (e) and HFD‐fed (f) WT and U9‐KO WAT to measure different myeloid cell populations by flow cytometry. Here, M1 macrophages (CD11c^+^), M2 macrophages (CD206^+^ or CD301^+^), adipose tissue‐resident macrophages (F4/80^+^), and monocytes (Ly6C^+^) expressed as percentages of the parent CD11b^+^ population are shown.

Adipose tissue contains numerous leukocyte populations in its stromal vascular cell (SVC) fraction, along with preadipocytes (Orr et al., 2013). During obesity, in addition to macrophages, other leukocytes, including neutrophils, monocytes, T cells, B cells, NK cells, etc., also infiltrate the adipose tissue, which plays an essential role in obesity‐associated inflammation (Boutens & Stienstra, 2016; Orr et al., 2013). Therefore, to identify if the increased *U90926* expression in HFD‐fed WT whole pWAT tissue RNA (Figure 5f) is associated with cells belonging to the SVC fraction (containing preadipocytes and immune cells) or to mature adipocytes, we separated SVC and mature adipocytes from pWAT (Figure 6a). We found that, in WT mice, *U90926* expression is significantly higher in the SVC fraction than in adipocytes (Figure 6b), with a 91% reduction in expression in SVC from U9‐KO mice as measured by RTq‐PCR to levels below the limit of reliable detection (Figure 6c). Since leukocyte infiltration into pWAT is a hallmark of obesity‐related inflammation, we have examined if *U90926* has any effect on regulating leukocyte infiltration in pWAT, focusing on the myeloid cell population, given our published findings on the function of *U90926* in macrophages (Sabikunnahar et al., 2023). We measured M1 macrophages (CD11c^+^), M2 macrophages (CD206^+^ or CD301^+^), adipose tissue‐resident macrophages (F4/80^+^), and monocytes (Ly6C^+^) as a percentage of CD11b^+^ cells in pWAT collected from both ND‐fed and HFD‐fed WT and U9‐KO mice by flow cytometry (Figure 6d). We found no differences in myeloid cell populations in pWAT of U9‐KO mice compared to WT, regardless of diet (Figure 6e, f). Altogether, these data suggest that *U90926* deletion in vivo lacks any effect on major phenotypes in diet‐induced obesity.

## 4 DISCUSSION

Obesity has become a global pandemic that affects over 1 billion people worldwide. It is a significant health risk associated with comorbidities including type 2 diabetes, nonalcoholic fatty liver disease, cardiovascular disease like hypertension, myocardial infarction, stroke, musculoskeletal disease (osteoarthritis), asthma, and some types of cancer, for example, breast, ovarian, prostate, liver, kidney, and colon (Blüher, 2019). Adipogenesis, or adipocyte differentiation, is an essential aspect of fat expansion in obesity (Ali et al., 2013). Therefore, understanding molecular mechanisms and triggers for adipogenesis during obesity is helpful in combating obesity. Many research groups have implicated lncRNAs as regulators of adipocyte differentiation (Sun et al., 2013; Wei et al., 2016). Chen et al. (2017) studied the lncRNA *U90926* in adipogenesis, showing that this gene *U90926 is* a negative regulator of adipogenesis using an in vitro 3T3‐L1 preadipocyte differentiation system and providing the first putative function for this novel lncRNA. Having previously generated the first (to our knowledge) mouse model deficient in *U90926* (Sabikunnahar et al., 2023), based on these findings, in this study, we investigated the contribution of *U90926* to weight gain and other obesity‐related phenotypes in vivo.

*U90926* was first reported and classified as lncRNA by a genome exploration research group (Okazaki et al., 2002). Since then, several groups, including ours, have characterized this lncRNA in distinct cell types, including 3T3‐L1 preadipocytes, microglia, mouse retinal cells, and macrophages, with distinct functional roles in each cell type (Chen et al., 2017, 2021; Sabikunnahar et al., 2023; Shirahama et al., 2020). We have previously reported that *U90926* is highly expressed in myeloid cells in a TLR‐Myd88‐p38 MAPK‐dependent manner and is protective during endotoxic shock (Sabikunnahar et al., 2023). We are also the first to identify that *U90926* can encode a small protein in its 264‐bp‐long open reading frame (ORF) (Sabikunnahar et al., 2023), suggesting that *U90926* may have a dual role in cellular processes as lncRNA and a protein‐coding gene. Here, we have evaluated the function of *U90926* in adiposity and weight gain using a knockout mouse model, U9‐KO, in which we deleted the whole genomic locus of *U90926*. In agreement with Chen et al. (2017), we found that *U90926* is expressed at detectable (albeit low) levels in visceral WAT and is highly expressed in 3T3‐L1 preadipocytes, with its expression decreasing as 3T3‐L1 cells become mature adipocytes (Figure 1a). However, in subsequent experiments, we found that the decrease in *U90926* expression in 3T3‐L1 cells is not dependent on the presence of adipocyte differentiation stimuli; instead, it may be the effect of prolonged cell culture, as undifferentiated 3T3‐L1 cells cultured in parallel showed the same expression pattern (Figure S2). This could be due to contact inhibition or other intrinsic factors that change gene expression patterns when cells are in culture for an extended period of time (Zaitseva et al., 2006).

Chen et al. (2017) showed that *U90926* represses adipogenesis in vitro, as siRNA‐mediated silencing of *U90926* caused increased expression of adipogenesis markers such as *Pparg*, *Cebpa*, *Fabp4*, and *Adipoq* and increased lipid accumulation during 3T3‐L1 differentiation, whereas overexpression of *U90926* had the opposite effect. Based on these findings, we expected that the U9‐KO mouse model would show increased adipogenesis in vivo. On the contrary, we have not found any major effect of *U90926* deficiency in vivo in terms of weight gain and other obesity‐related phenotypes, such as fat accumulation, metabolic changes including plasma levels of glucose, TG, NEFA, and insulin, WAT histology, and adipogenesis molecular markers (Figures 2, 3, 4). Leptin concentrations were slightly decreased in male but not female U9‐KO mice (Figure 3h); however, their food intake (evaluated in GMC; data not shown) and other energy metabolism data did not correlate with this finding, indicating unaltered leptin signaling in U9‐KO mice (Harris, 2014). Generally, the 3T3‐L1 differentiation system is widely used for studying adipogenesis because of its uniform morphological and biochemical nature, comparable with adipocytes in vivo (Novikoff et al., 1980). However, the drawback of this model is that molecular events of adipogenesis in vitro may not fully reflect adipogenesis in vivo, where different other cell types, including adipose tissue macrophages, are also intimately involved (Ntambi & Young‐Cheul, 2000). Our data echo this and suggest that in vitro adipogenesis phenotypes may not be totally reflective of adipogenesis phenomena in vivo. Alternatively, the contribution of *U90926* in adipose tissue homeostasis may be redundant and may be compensated by other genes or pathways, although a lack of significant gene expression changes in WAT as seen in our transcriptional profiling experiments argues against this possibility.

Since the deletion of *U90926* in vivo was insufficient to change any phenotypes related to obesity in mice fed standard chow, we stressed the system to robustly induce obesity using an HFD. Interestingly, we found that, compared with ND, HFD increased *U90926* expression in whole WAT (Figure 5f), which contradicts the findings of Chen et al. (2017). Nonetheless, and as with ND, we have found no effect of the *U90926* deletion in HFD‐fed mice on weight gain, fat accumulation, adipogenesis markers in the pWAT, and leukocyte infiltration into the pWAT (Figures 5 and 6), although we acknowledge that, unlike the normal diet studies, measurements of in vivo fat mass or additional metabolic parameters were not performed in our high‐fat diet studies.

The role of adipogenesis in regulating obesity phenotypes is controversial in the field. Some studies showed that adipogenesis increases significantly during both genetic and diet‐induced obesity (Faust et al., 1978, 1984; Hirsch & Batchelor, 1976). Others have shown that adipocyte hypertrophy is the main contributor to obesity (Hirsch & Han, 1969; Johnson & Hirsch, 1972). Recent studies revealed that increased adipogenesis has paradoxically beneficial metabolic outcomes in obesity (Ghaben & Scherer, 2019; Jakab et al., 2021). Thus, adipogenesis regulation in vitro may not be an indicator of obesity phenotypes in vivo. Regardless, the lack of changes in adipocyte morphology in pWAT and adipogenesis marker expression in U9‐KO compared to WT (Figure 4) in our studies suggests *U90926* deletion does not affect adipogenesis or other obesity phenotypes in vivo. We note that while we did establish the loss of *U90926* expression in whole adipose tissue from U9‐KO animals (Figure 1a), in isolated WT adipocytes, the expression level of *U90926* RNA is near the limit of detection/background (Figure 6b), and hence we were unable to demonstrate downregulation in KO adipocytes. However, we were able to show the loss of *U90926* expression in SVC, collected from U9‐KO adipose tissue, compared to WT adipose tissue (Figure 6c). The low expression of *U90926* in mature adipocytes is expected, since Chen et al. (2017) showed that *U90926* expression decreases greatly during adipocyte maturation in vitro, as well as at the whole adipose tissue level during obesity in vivo.

Besides adipocytes and preadipocytes, WAT tissue comprises many different cell types that influence adipogenesis and adipocyte activity in vivo (Boutens & Stienstra, 2016; Orr et al., 2013). Adipose tissue macrophages, in particular, play a vital role in adipogenesis and metabolic changes during obesity (Cheng et al., 2019), and we have shown previously that *U90926* is highly expressed in LPS‐activated macrophages and even in whole WAT following systemic LPS administration in vivo (Sabikunnahar et al., 2023). In addition, our data show that *U90926* is expressed in stromal vascular cells (SVCs) instead of in mature adipocytes (Figure 6b), which indicates an increased *U90926* expression signal in HFD‐SVC may come from macrophages during obesity, induced by chronic inflammatory signals or cell‐damage‐associated molecular patterns. Thus, there could be a macrophage‐driven *U90926* role in obesity and/or sepsis‐related adipose tissue responses that can be explored in future studies. Alternatively, it is also likely that the *U90926* expression in the SVC comes from preadipocytes, which are also present in this cellular fraction and would be expected to express *U90926* (Chen et al., 2017). A conditional knockout model specific to macrophage or adipocyte lineage could be used to further determine the contribution of *U90926* to obesity in vivo, with temporally inducible models to account for any developmental compensatory effects on loss of *U90926* expression. Nonetheless, our present results demonstrate that loss of *U90926* does not affect obesity‐related phenotypes in mice under a normal or high‐fat diet condition, suggesting that unlike its role in myeloid cell‐mediated inflammation, this gene may not have an essential role in adipocyte biology.

## FUNDING INFORMATION

This work was supported by grants from the National MS Society (research grant RR‐1602‐07780) and the NIH NIAID (R21AI151116) to DNK. The GMC study was supported by the German Federal Ministry of Education and Research (Infrafrontier grant 01KX1012 to MHdA) and the German Center for Diabetes Research (DZD) (MHdA). The authors would like to thank Animal care management (OCAM) at the UVM, Dr. Roxana del Rio‐Guerra for the flow cytometry and cell sorting facility, and Dr. Cory Teuscher (Department of Medicine, UVM) for many helpful discussions and feedback.

## ETHICS STATEMENT

All animal experiments performed at the University of Vermont (UVM) were approved by the UVM Animal Care and Use Committee (IACUC; protocol number X1‐061). All guidelines for ethical protocols and care of experimental animals established by the National Institutes of Health (NIH) were followed. At the German Mouse Clinic (GMC), animals were maintained in individually ventilated cages with water and standard mouse chow according to the directive 2010/63/EU, German national laws, and GMC housing conditions (www.mouseclinic.de). All tests performed were approved by the responsible authority of the district government of Upper Bavaria. We also confirm that we understand the ethical principles under which the journal operates and that our work complies with the animal ethics checklist as outlined in the instructions to authors.

## Supporting information


Data S1.

Figure S2.

Figure S3.
