# Supplementary material for: LncRNA U90926 is dispensable for the development of obesity‐associated phenotypes in vivo
Source: Physiol Rep. 2024 Jan 3;12(1):e15901. doi: 10.14814/phy2.15901 (PMC10764201; doi:10.14814/phy2.15901)
Supplement: Supplementary file 1 — Data S1. [file PHY2-12-e15901-s002.pdf]

Supplemental phenotyping data:
Mouse line U90926 (cDNA sequence U90926)

In this screening approach more than 500 parameters were measured and no correction for multiple testing was performed. Data is analyzed using automated R-scripts. Statistical analysis was dependent on the data (ANOVA, Linear Model, Wilcoxon rank-sum test as appropriate).

https://www.mouseclinic.de/about-gmc/the-arrive-essential-10/index.html

Distance travelled

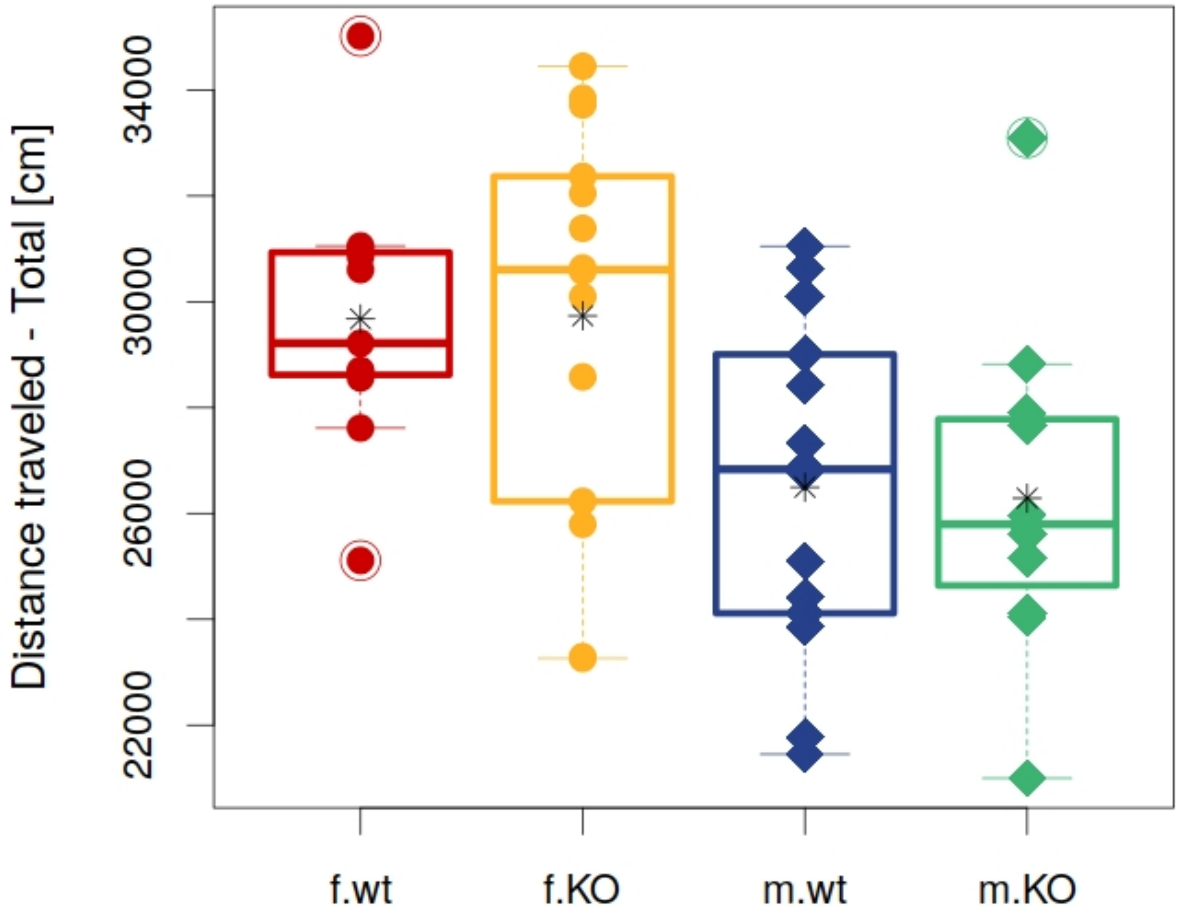

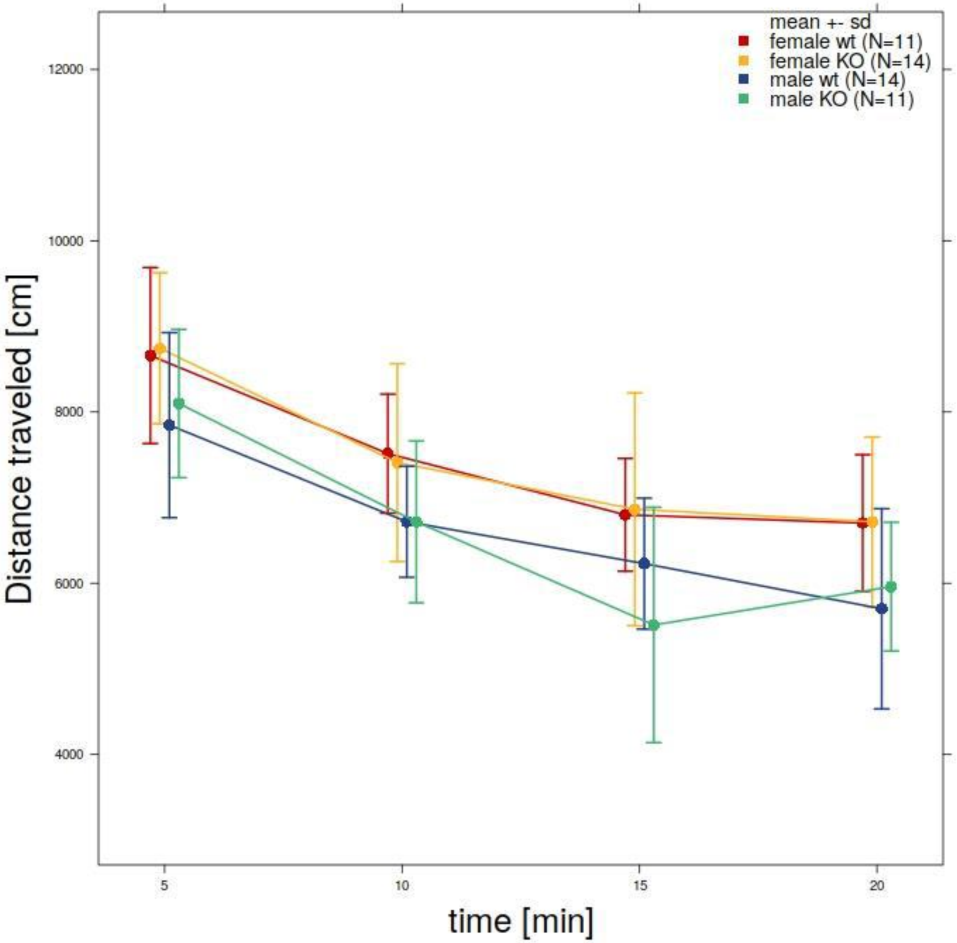

No genotype effect on distance travelled

Rearing activity

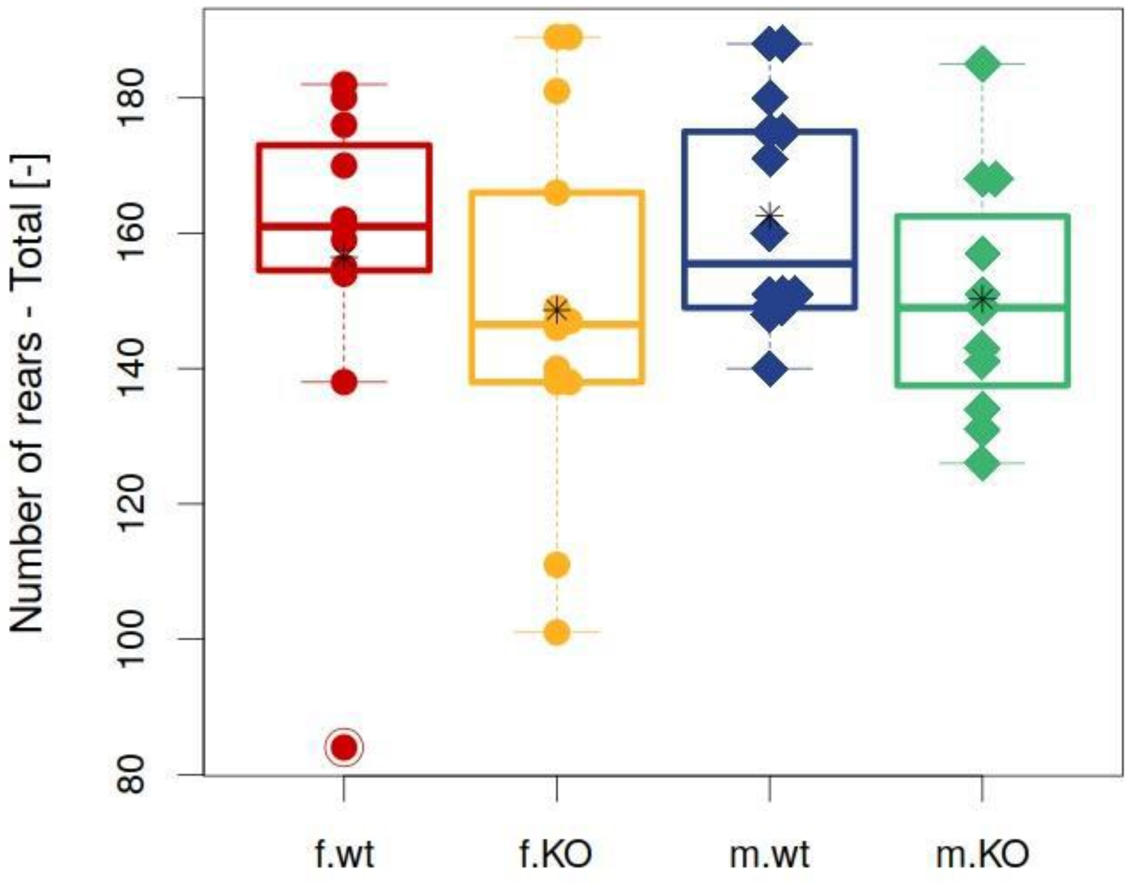

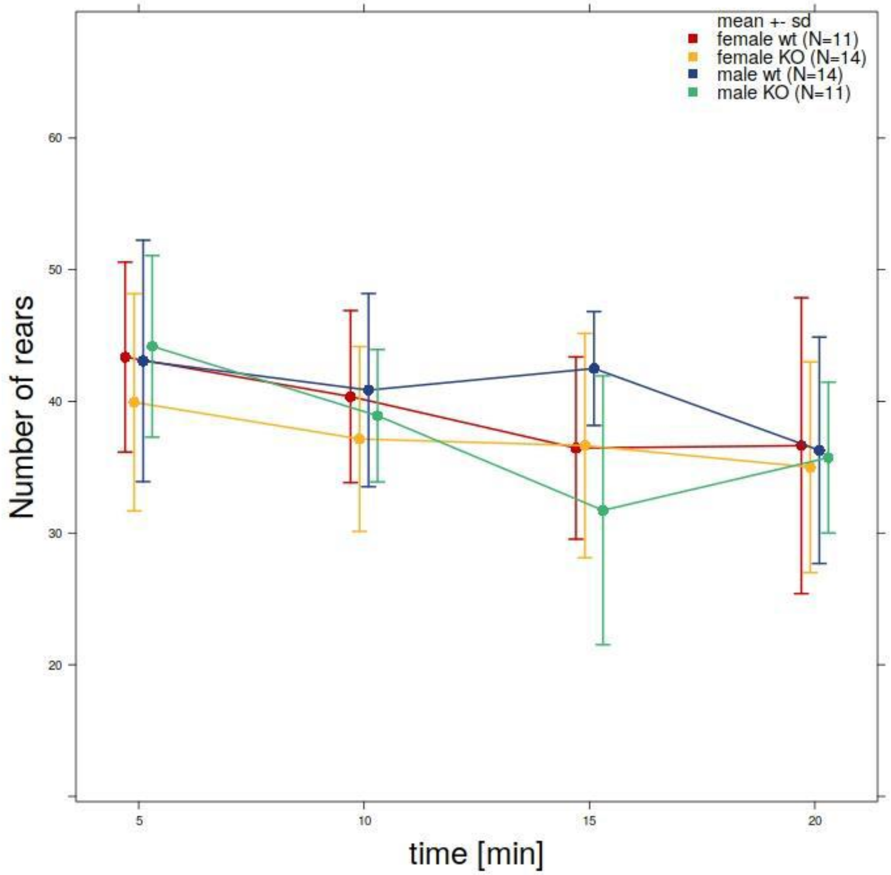

No genotype effect on rearing activity

Time spent and distance in centre

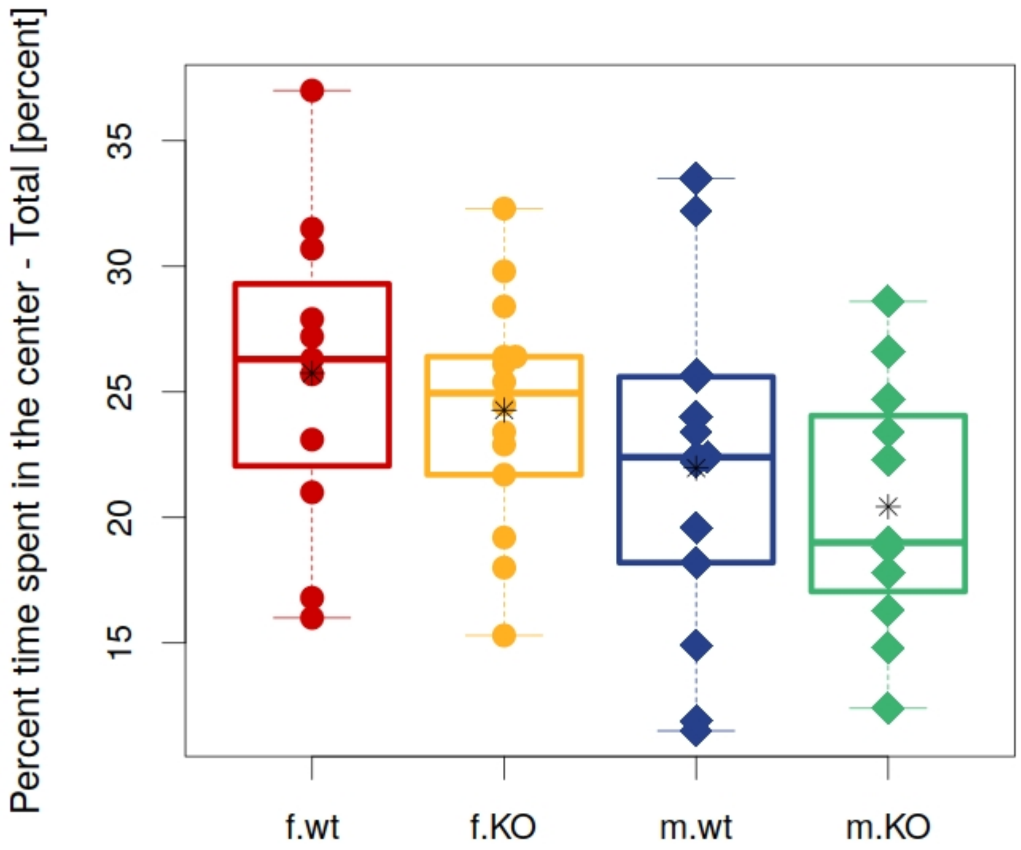

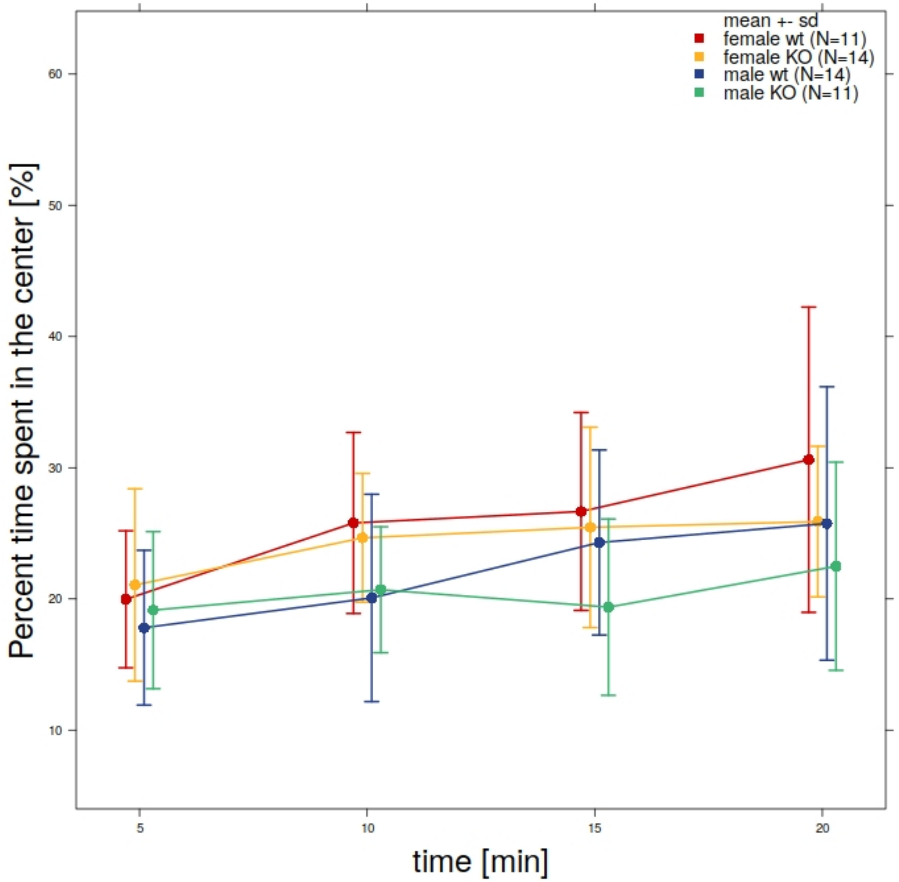

No genotype effect on centre time

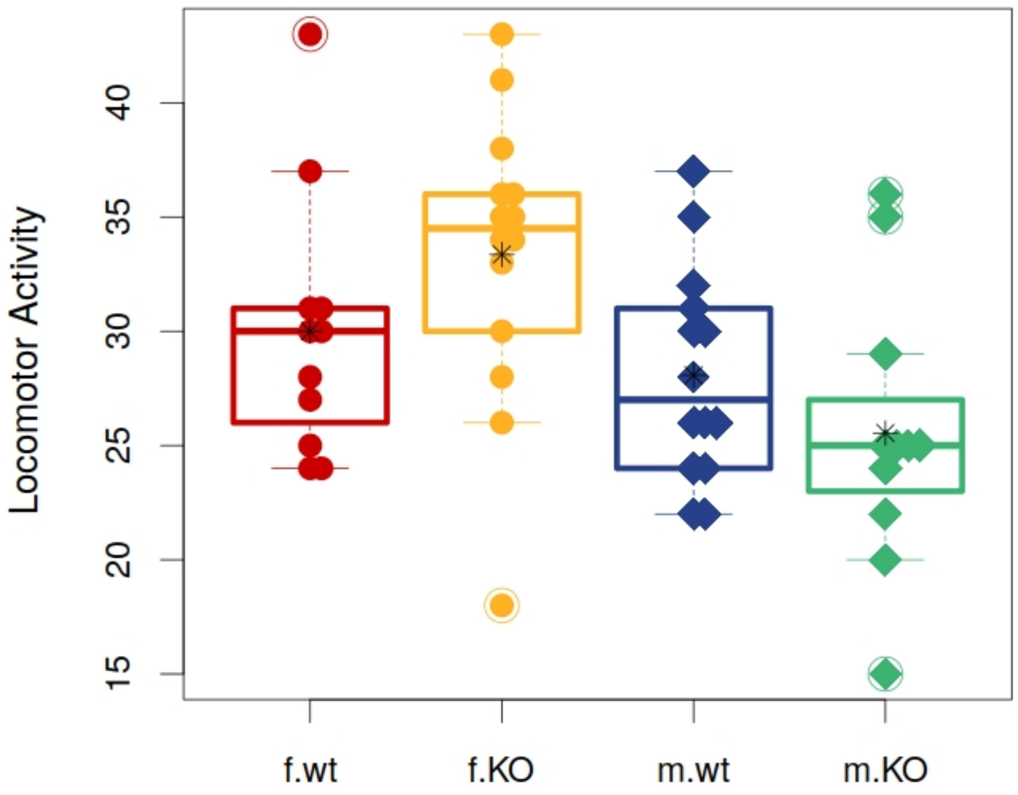

No difference for any SHIRPA parameter

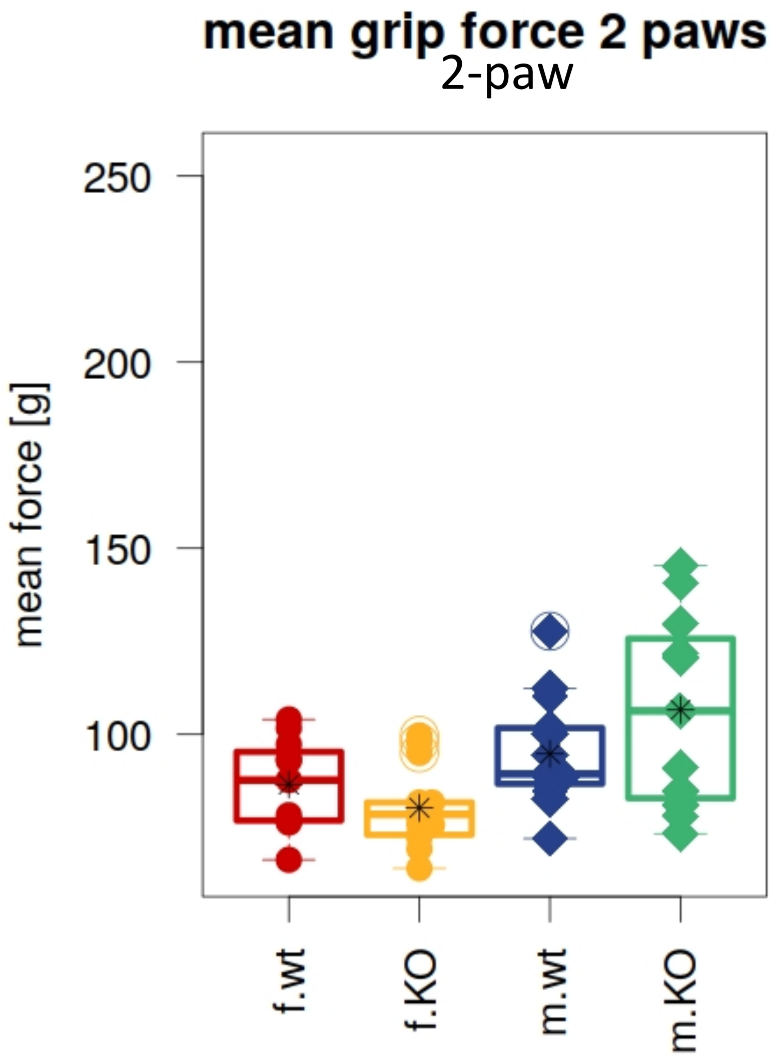

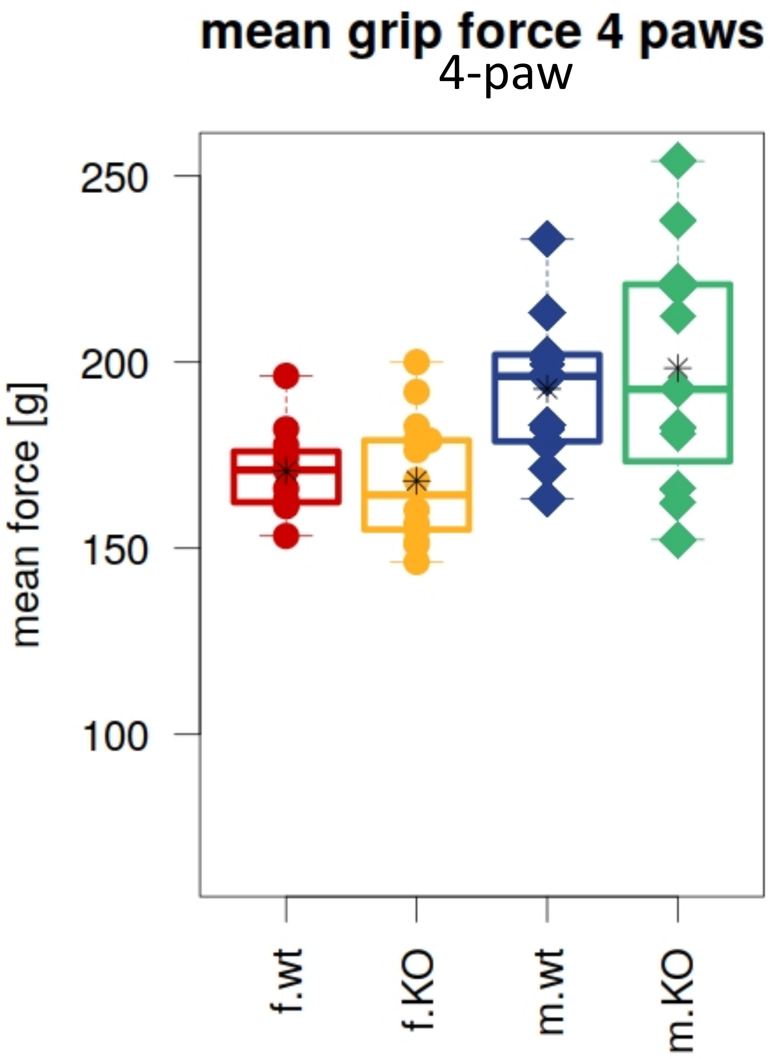

No genotype-related differences at grip strength

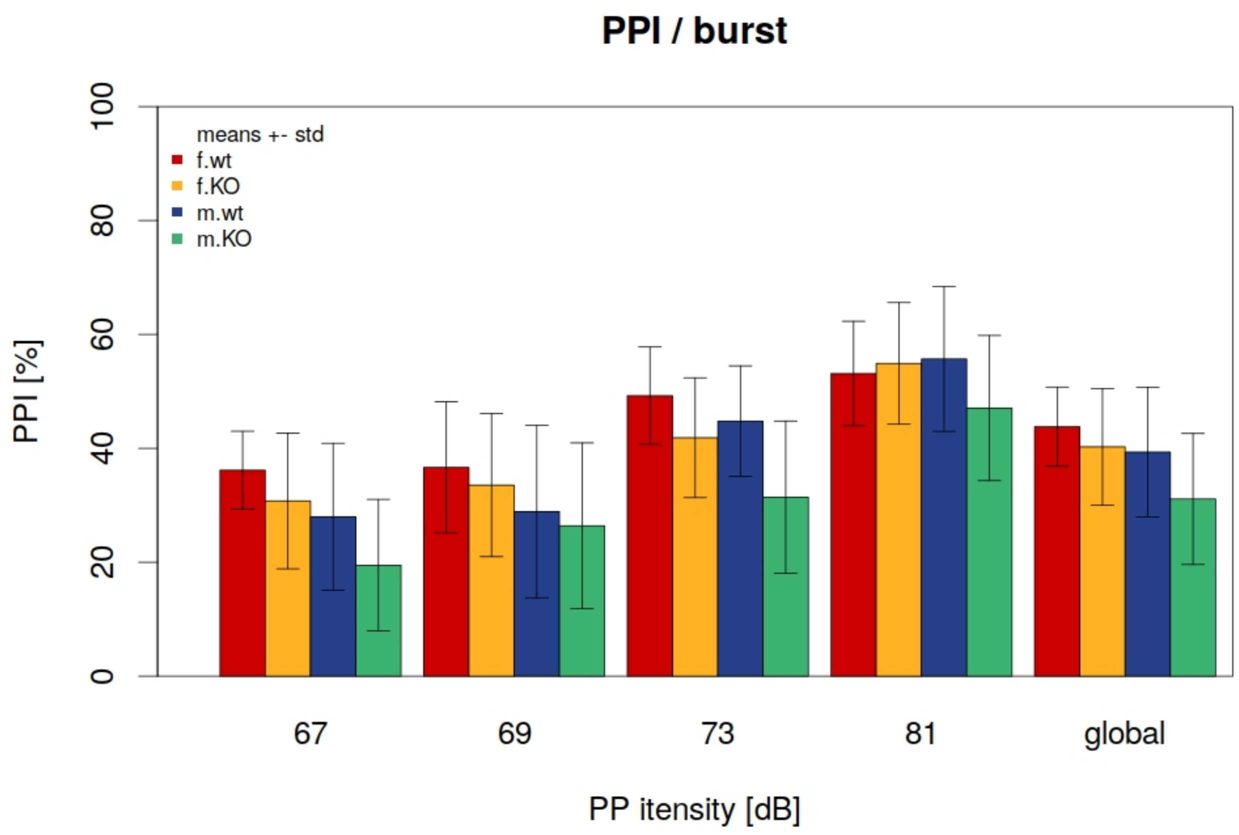

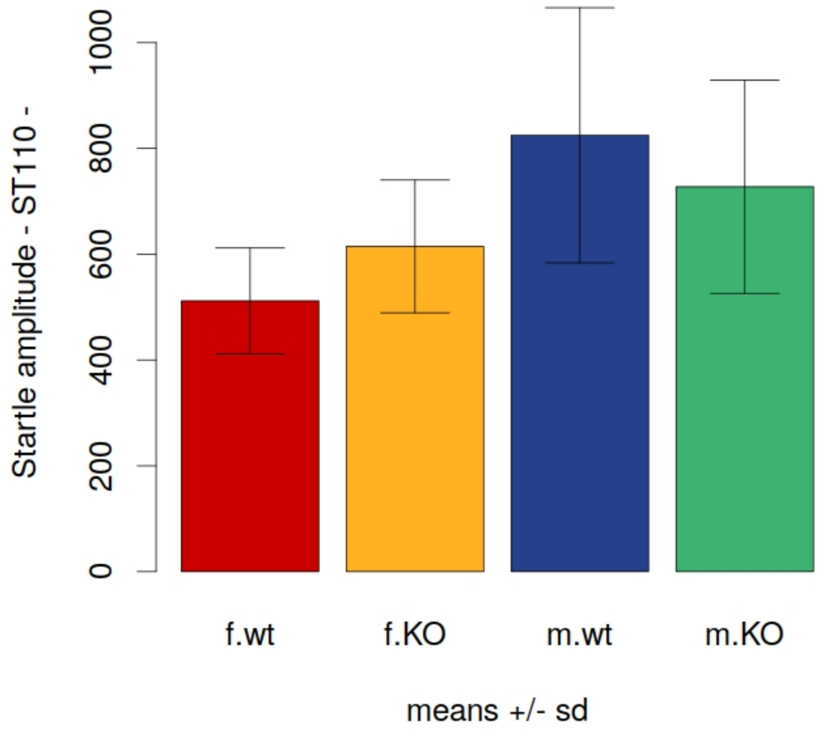

Slightly decreased prepulse inhibition
(*67 dB p = 0.035, 73 dB p = 0.001*) and
No significant effects on acoustic startle

First reaction latencies

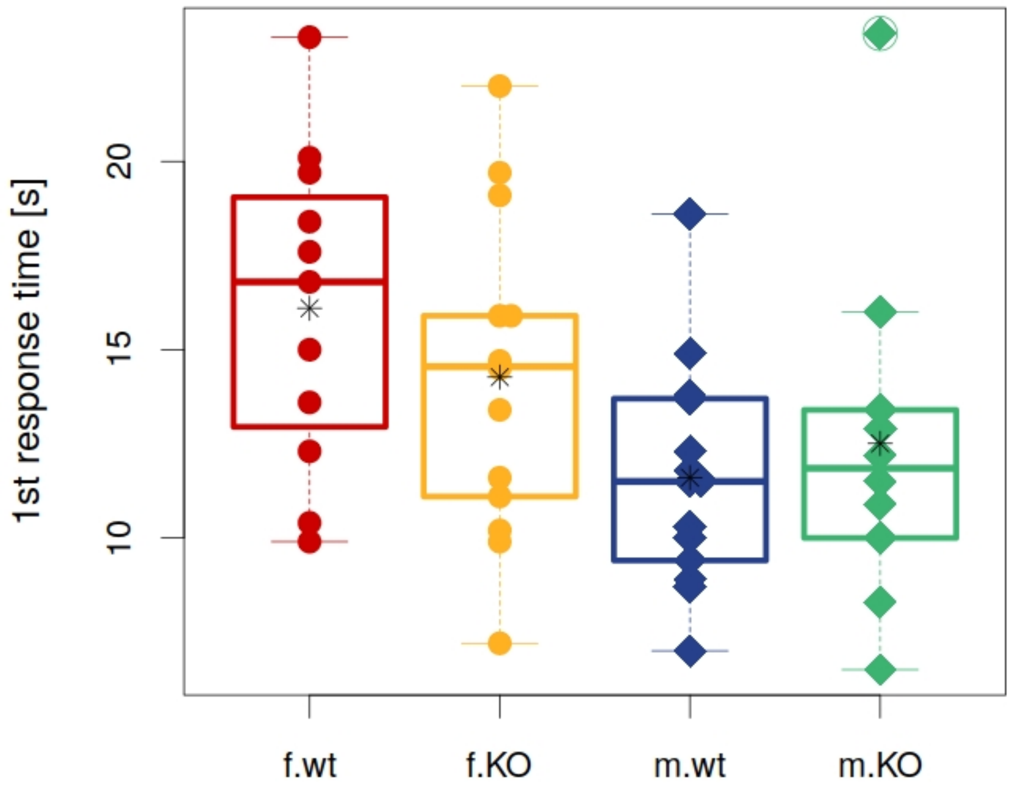

Second reaction latencies

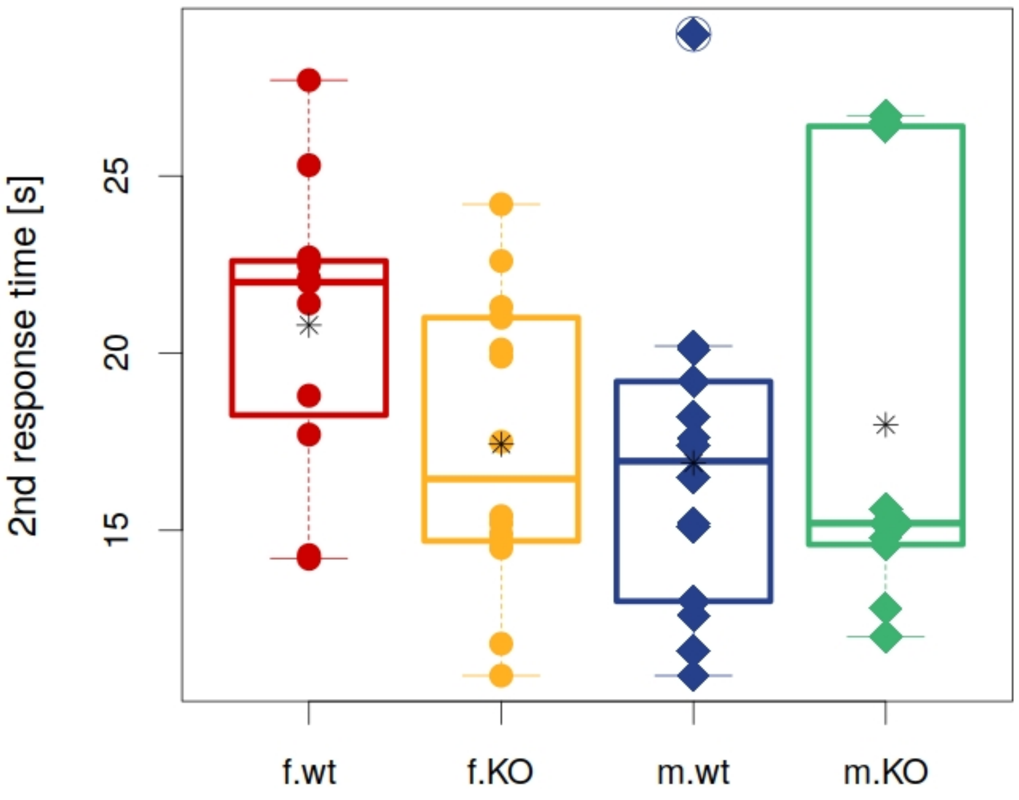

No genotype effect on hotplate latencies or types or reactions (not shown)

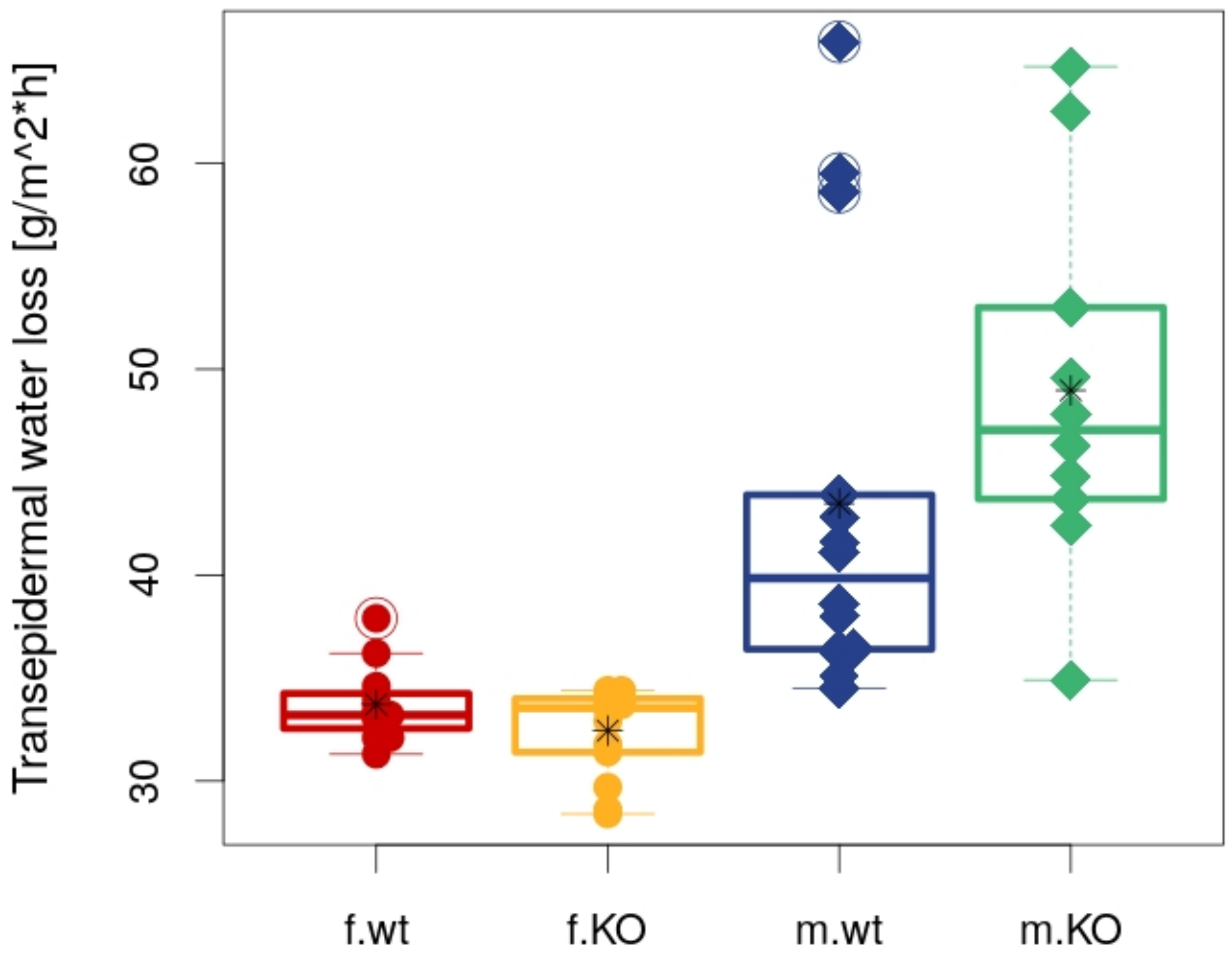

No significant differences in the TEWL, values are in the normal range of WT mice

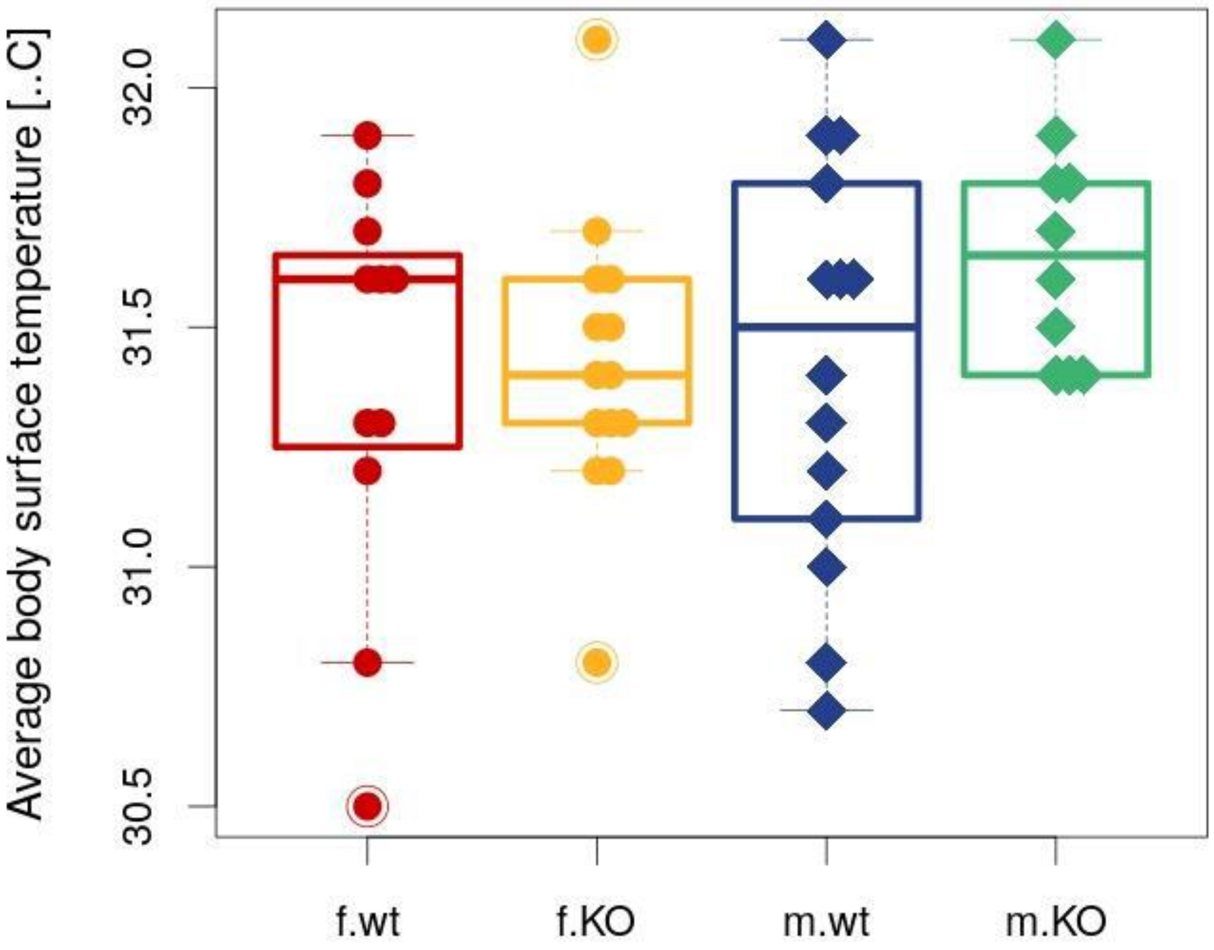

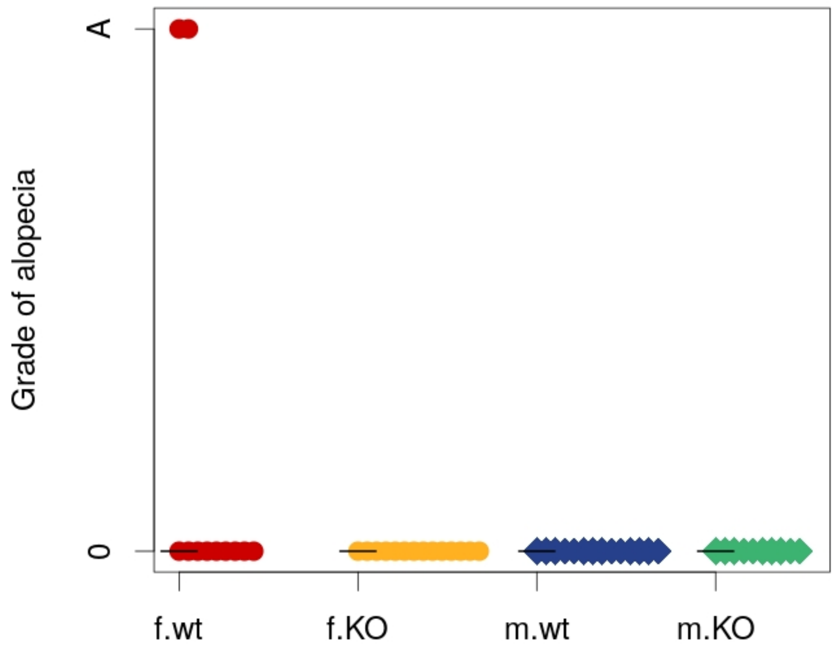

No differences in the body surface temperature or alopecia degree

Body composition analysis I (13 weeks old)

	female		male		linear model	linear model	linear model	linear model
	wt	KO	wt	KO	sex	genotype	Bodymass	sex:genotype
	n=11	n=14	n=14	n=10				
	mean ± sd	mean ± sd	mean ± sd	mean ± sd	p-value	p-value	p-value	p-value
**Body mass NMR**	21.7 ± 0.8	21.1 ± 1	28.9 ± 1.5	28.4 ± 1.9	< 0.001	0.143	NA	0.873
**Fat mass NMR**	4.7 ± 0.5	4.6 ± 0.4	6.2 ± 0.7	6 ± 0.7	0.022	0.805	< 0.001	0.478
**Lean mass NMR**	13.8 ± 0.5	13.3 ± 0.5	18.9 ± 1	18.8 ± 1.2	0.002	0.969	< 0.001	0.4
**Free fluid**	0.8 ± 0.1	0.9 ± 0.2	0.9 ± 0.1	0.8 ± 0.1	0.001	0.769	< 0.001	0.116
**Adiposity Index**	0.3 ± 0	0.3 ± 0	0.3 ± 0	0.3 ± 0	0.004	0.764	0.022	0.321

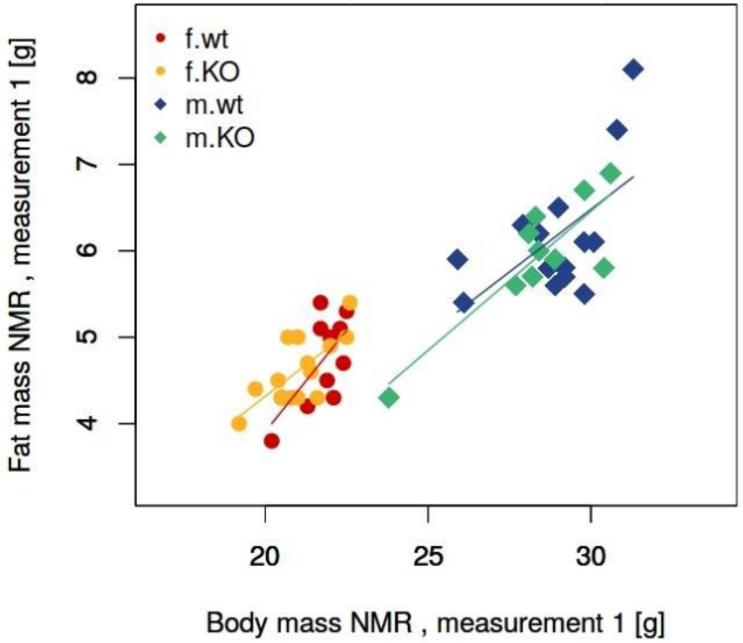

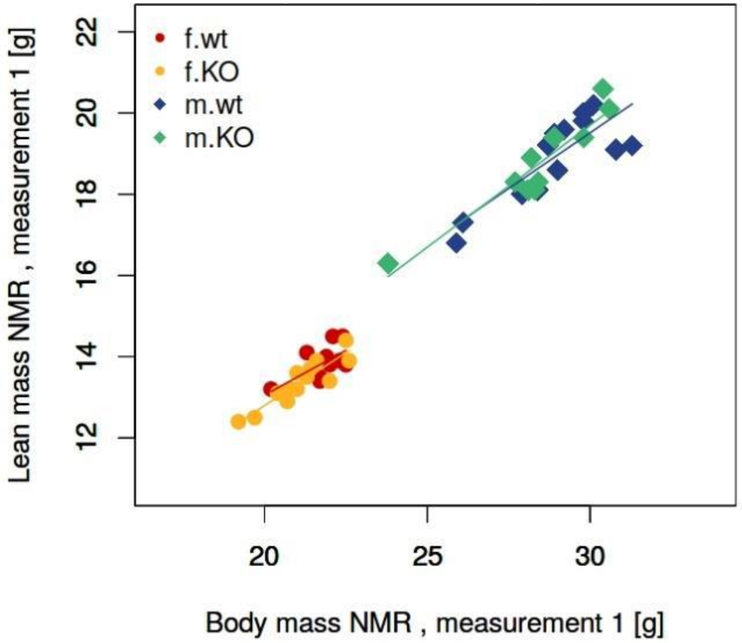

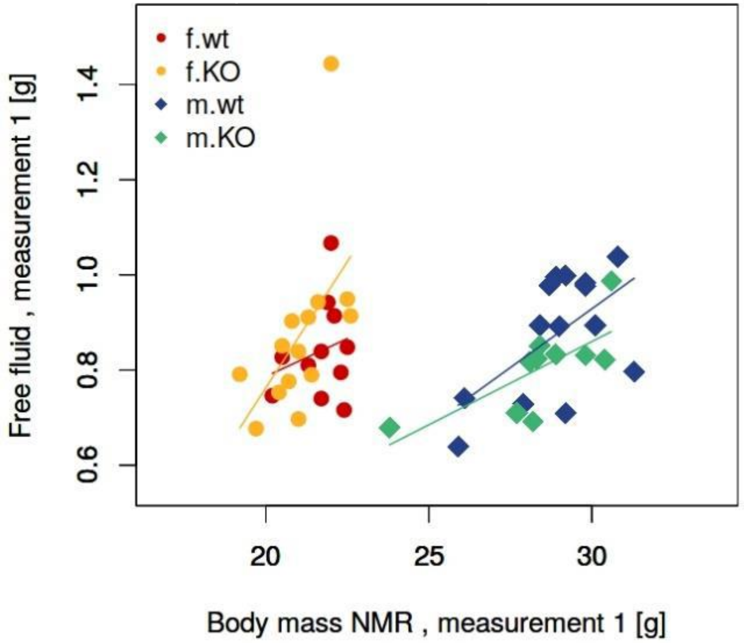

Body composition analysis I (13 weeks old)

	female		male		linear model	linear model	linear model	linear model
	wt	KO	wt	KO	sex	genotype	Bodymass	sex:genotype
	n=11	n=14	n=14	n=10				
	mean ± sd	mean ± sd	mean ± sd	mean ± sd	p-value	p-value	p-value	p-value
**Body mass NMR**	21.7 ± 0.8	21.1 ± 1	28.9 ± 1.5	28.4 ± 1.9	< 0.001	0.143	NA	0.873
**Fat mass NMR**	4.7 ± 0.5	4.6 ± 0.4	6.2 ± 0.7	6 ± 0.7	0.022	0.805	< 0.001	0.478
**Lean mass NMR**	13.8 ± 0.5	13.3 ± 0.5	18.9 ± 1	18.8 ± 1.2	0.002	0.969	< 0.001	0.4
**Free fluid**	0.8 ± 0.1	0.9 ± 0.2	0.9 ± 0.1	0.8 ± 0.1	0.001	0.769	< 0.001	0.116
**Adiposity Index**	0.3 ± 0	0.3 ± 0	0.3 ± 0	0.3 ± 0	0.004	0.764	0.022	0.321

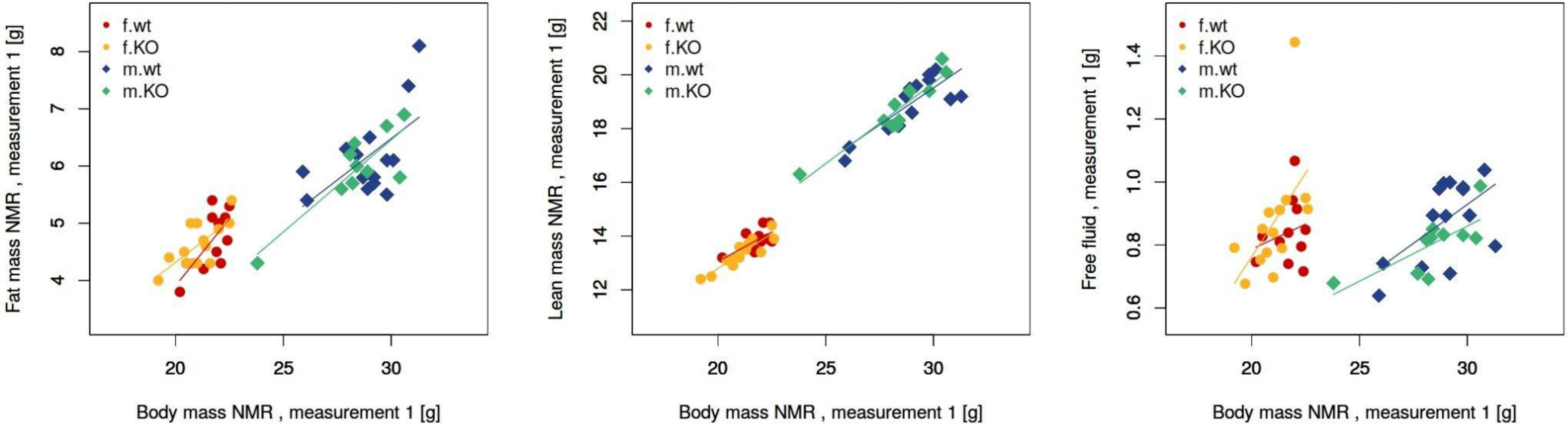

No differences concerning body composition parameters

	female		male		Linear model	Linear model	Linear model	Linear model
	wt	KO	wt	KO	sex	genotype	body mass	sex:genotype
	n=11	n=14	n=14	n=10				
	mean ± sd	mean ± sd	mean ± sd	mean ± sd	p-value	p-value	p-value	p-value
**Avg. body weight [g]**	21.3 ± 0.8	20.5 ± 0.7	27.9 ± 1.6	27.6 ± 1.8	< 0.001	0.12	NA	0.503
**Body weight loss [Δ g]**	0.13 ± 0.48	0.3 ± 0.51	0.44 ± 0.43	0.41 ± 0.39	0.12	0.581	NA	0.457
**Food intake [g]**	2.7 ± 0.4	2.6 ± 0.5	2.8 ± 0.6	2.8 ± 0.4	0.156	0.851	0.074	0.647
**Avg. VO2 [ml/(h animal)]**	83.504 ± 3.986	84.734 ± 4.8	98.605 ± 5.223	97.446 ± 4.061	0.254	0.493	0.006	0.218
**Min. VO2 [ml/(h animal)]**	62.273 ± 3.663	62.429 ± 4.751	71.929 ± 5.313	72.6 ± 4.427	0.122	0.594	0.309	0.93
**Max. VO2 [ml/(h animal)]**	110.364 ± 3.88	115.714 ± 9.73	132.357 ± 8.205	129.1 ± 5.744	0.978	0.213	0.002	0.017
**Avg. RER VCO2/VO2**	0.877 ± 0.014	0.88 ± 0.023	0.867 ± 0.02	0.868 ± 0.019	0.052	0.739	NA	0.854
**Avg. heat [kJ/(h animal)]**	1.718 ± 0.081	1.744 ± 0.1	2.024 ± 0.113	2 ± 0.083	< 0.001	0.964	NA	0.376
**Avg. distance [cm]**	2945 ± 653	3792 ± 2446	2976 ± 579	2981 ± 830	0.352	0.309	NA	0.315
**Avg. rearing [counts]**	135 ± 82	142 ± 58	123 ± 60	88 ± 33	0.064	0.414	NA	0.23

Indirect Calorimetry (13 weeks old) – Results Overview

	female		male		Linear model	Linear model	Linear model	Linear model
	wt	KO	wt	KO	sex	genotype	body mass	sex:genotype
	n=11	n=14	n=14	n=10				
	mean ± sd	mean ± sd	mean ± sd	mean ± sd	p-value	p-value	p-value	p-value
**Avg. body weight [g]**	21.3 ± 0.8	20.5 ± 0.7	27.9 ± 1.6	27.6 ± 1.8	< 0.001	0.12	NA	0.503
**Body weight loss [Δ g]**	0.13 ± 0.48	0.3 ± 0.51	0.44 ± 0.43	0.41 ± 0.39	0.12	0.581	NA	0.457
**Food intake [g]**	2.7 ± 0.4	2.6 ± 0.5	2.8 ± 0.6	2.8 ± 0.4	0.156	0.851	0.074	0.647
**Avg. VO2 [ml/(h animal)]**	83.504 ± 3.986	84.734 ± 4.8	98.605 ± 5.223	97.446 ± 4.061	0.254	0.493	0.006	0.218
**Min. VO2 [ml/(h animal)]**	62.273 ± 3.663	62.429 ± 4.751	71.929 ± 5.313	72.6 ± 4.427	0.122	0.594	0.309	0.93
**Max. VO2 [ml/(h animal)]**	110.364 ± 3.88	115.714 ± 9.73	132.357 ± 8.205	129.1 ± 5.744	0.978	0.213	0.002	0.017
**Avg. RER VCO2/VO2**	0.877 ± 0.014	0.88 ± 0.023	0.867 ± 0.02	0.868 ± 0.019	0.052	0.739	NA	0.854
**Avg. heat [kJ/(h animal)]**	1.718 ± 0.081	1.744 ± 0.1	2.024 ± 0.113	2 ± 0.083	< 0.001	0.964	NA	0.376
**Avg. distance [cm]**	2945 ± 653	3792 ± 2446	2976 ± 579	2981 ± 830	0.352	0.309	NA	0.315
**Avg. rearing [counts]**	135 ± 82	142 ± 58	123 ± 60	88 ± 33	0.064	0.414	NA	0.23

No differences concerning energy metabolism

Results electrocardiography in U90926 wt versus KO mice (15 weeks)

	female		male		female	male	overall
	wt	KO	wt	KO			
	n=11	n=13	n=14	n=10			
	median [25%, 75%]	median [25%, 75%]	median [25%, 75%]	median [25%, 75%]	p-value	p-value	p-value
**Heart Rate [bpm]**	762.4 [749.5 , 773.9]	752.5 [745 , 759.3]	756.5 [752.7 , 768.5]	755.4 [748.9 , 768.2]	0.186	0.875	0.208
**RR [ms]**	78.7 [77.5 , 80]	79.8 [79 , 80.5]	79.3 [78 , 79.8]	79.5 [78.1 , 80.2]	0.163	0.852	0.183
**PR [ms]**	30.2 [28.9 , 30.6]	30.1 [29.3 , 30.7]	29.9 [28.5 , 32.9]	31 [29.6 , 32.1]	0.876	0.784	0.577
**QRS [ms]**	13.7 [12.3 , 14.5]	12.7 [12.2 , 13.2]	12.7 [11.7 , 13.7]	12.8 [12.2 , 13.3]	0.171	0.655	0.223
**QT [ms]**	22.9 [22.1 , 23.9]	21.6 [20.8 , 22.3]	21.4 [19.6 , 23.4]	21.9 [20 , 22.6]	0.079	0.989	0.303
**QTc [ms]**	81.9 [79.4 , 84.5]	76.2 [72.9 , 79.3]	75.8 [69.5 , 83.7]	77.7 [70.5 , 81]	0.029	1	0.205
**QT dispersion [ms]**	1.9 [1.2 , 2.6]	0.9 [0.4 , 1.3]	1.1 [0.7 , 3.5]	1.2 [0.7 , 2.2]	0.09	0.516	0.086
**QTc dispersion [ms]**	6.6 [4 , 9.8]	3.2 [1.2 , 4.8]	4 [2.7 , 12.6]	4.5 [2.6 , 7.8]	0.13	0.463	0.094
**P [ms]**	10.2 [9.6 , 12.4]	10.1 [8.9 , 11.9]	11.2 [9.9 , 13.4]	11.3 [10.7 , 12.2]	0.433	0.886	0.477

Data presented in medians and confidence intervals. Statistics: Wilcoxon Rank Sum Test

Results echocardiography in U90926 wt versus KO mice

	female		male		female	male	overall
	wt	KO	wt	KO			
	n=11	n=14	n=14	n=10			
	Median [25%, 75%]	Median [25%, 75%]	Median [25%, 75%]	Median [25%, 75%]	p-value	p-value	p-value
**IVSs [mm]**	0.55 [0.55 , 0.56]	0.55 [0.55 , 0.55]	0.55 [0.53 , 0.58]	0.54 [0.52 , 0.56]	0.909	0.33	0.474
**IVSd [mm]**	0.54 [0.53 , 0.56]	0.54 [0.53 , 0.55]	0.55 [0.53 , 0.56]	0.53 [0.53 , 0.54]	0.841	0.199	0.314
**LVPWs [mm]**	0.56 [0.54 , 0.57]	0.55 [0.52 , 0.56]	0.55 [0.55 , 0.58]	0.55 [0.53 , 0.56]	0.36	0.376	0.178
**LVPWd [mm]**	0.55 [0.55 , 0.56]	0.55 [0.54 , 0.56]	0.57 [0.54 , 0.58]	0.54 [0.53 , 0.55]	0.729	0.146	0.121
**LVIDs [mm]**	1.22 [1 , 1.7]	1.62 [1.42 , 1.96]	1.87 [1.2 , 2.31]	1.58 [1.29 , 1.87]	0.217	0.785	0.666
**LVIDd [mm]**	2.74 [2.5 , 3.08]	3.12 [2.96 , 3.35]	3.31 [2.79 , 3.78]	3.27 [2.95 , 3.54]	0.066	0.698	0.418
**Weight [g]**	23.1 [22.1 , 23.4]	22.3 [21.4 , 22.9]	30 [29.3 , 30.9]	29.1 [28.4 , 29.6]	0.298	0.149	0.081
**Heart rate Echo [bpm]**	486.01 [397.31 , 588.1]	380.13 [303.35 , 445.37]	416.04 [308.13 , 568.89]	467.09 [418.45 , 644.54]	0.12	0.285	0.684
**Respiration Rate Echo [1/min]**	200 [183.22 , 217.31]	193.56 [178.16 , 200.85]	231.89 [199.18 , 241.22]	237.62 [220.78 , 250.7]	0.366	0.24	0.725
**Fractional shortening [percent]**	53.1 [47.84 , 61.62]	49.4 [42.34 , 57.45]	45.94 [41.03 , 57.66]	50.34 [42.7 , 57.37]	0.344	0.977	0.656
**Ejection fraction [percent]**	85.89 [80.38 , 91.6]	81.73 [74.6 , 89.15]	78.06 [72.65 , 88.97]	82.93 [75.21 , 88.18]	0.344	0.931	0.641
**Left ventricular mass corrected [mg]**	33.06 [25.95 , 37.35]	41.94 [35.91 , 46.22]	46.41 [33.88 , 53.45]	42.66 [34.51 , 46.51]	0.044	0.395	0.555
**Stroke Volume [mul]**	24.01 [19.11 , 28.61]	32.51 [27.43 , 34.6]	37.17 [26 , 41.05]	33.9 [29.9 , 41.72]	0.051	0.666	0.572
**Cardiac output [ml/min]**	10.599 [9.832 , 13.82]	10.574 [8.804 , 12.595]	15.072 [10.261 , 17.81]	14.857 [12.578 , 16.595]	0.647	0.666	0.913
**Left ventricular mass [mg]**	39.71 [32.45 , 44.72]	47.09 [42.08 , 51.69]	53.9 [40.93 , 61.06]	51.48 [41.61 , 56.92]	0.075	0.508	0.529

Data presented in medians and confidence intervals. Statistics: Wilcoxon Rank Sum Test

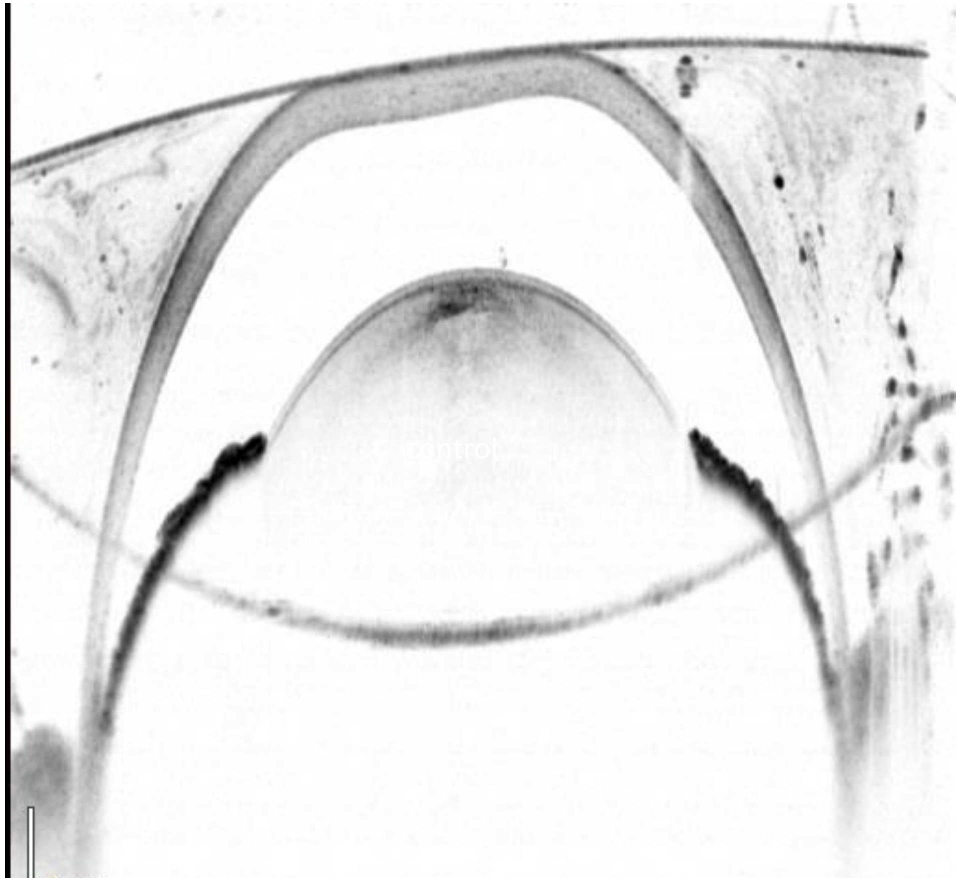

wild type

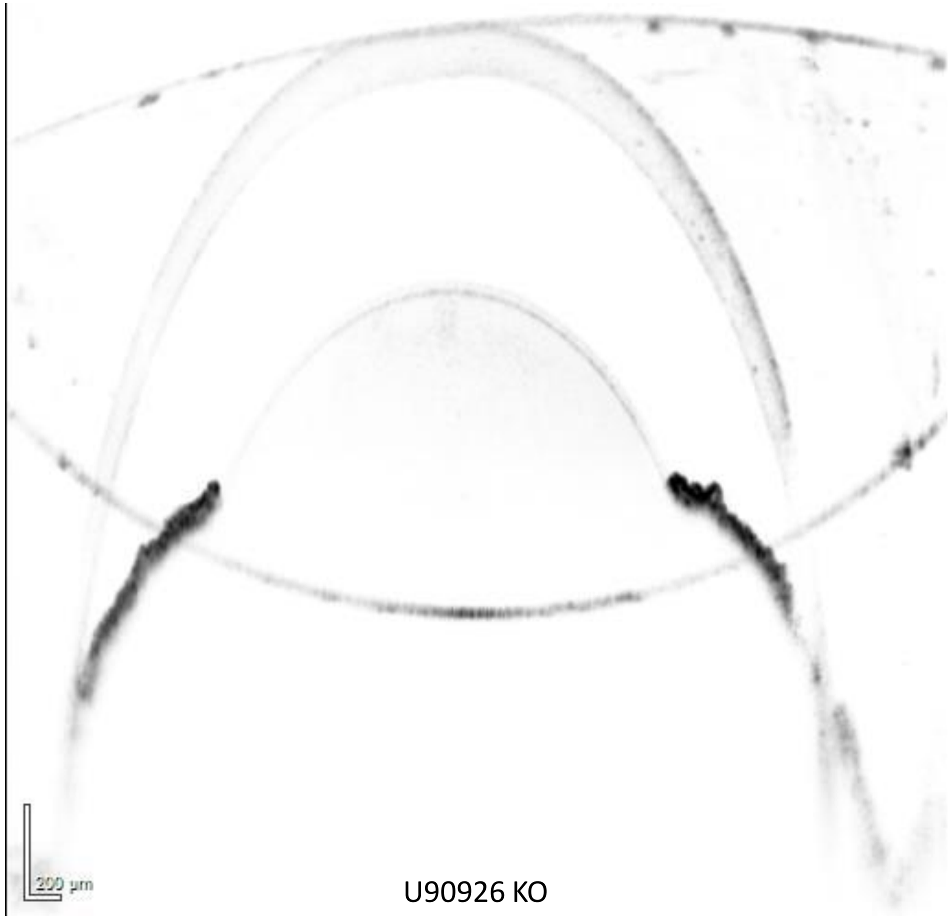

U90926 KO

Lens and cornea are developed properly.

Results eye morphology (16 weeks)

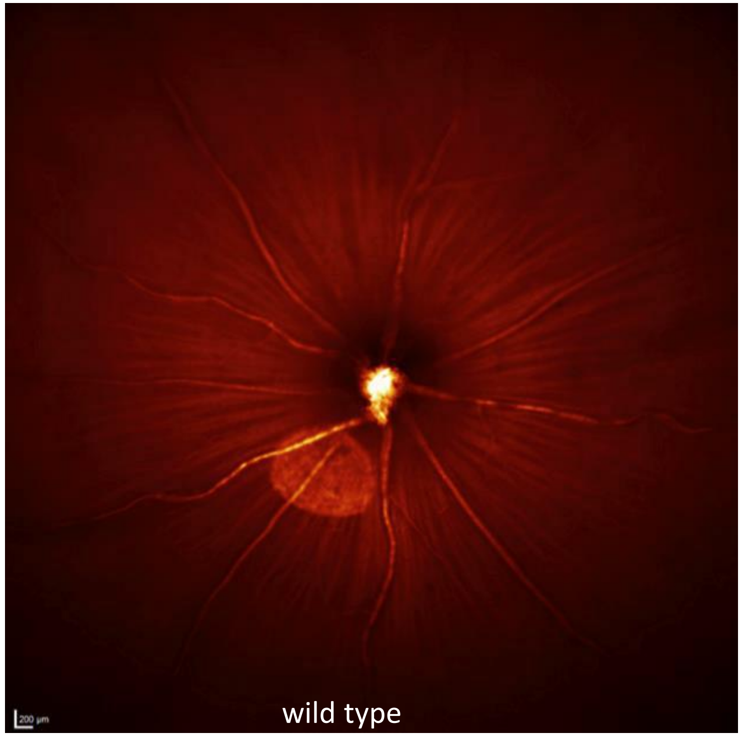

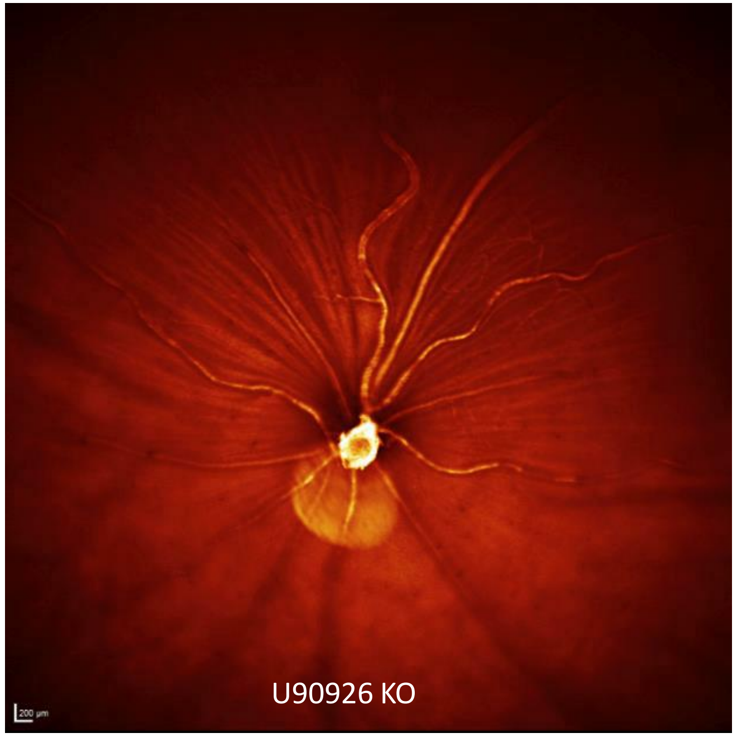

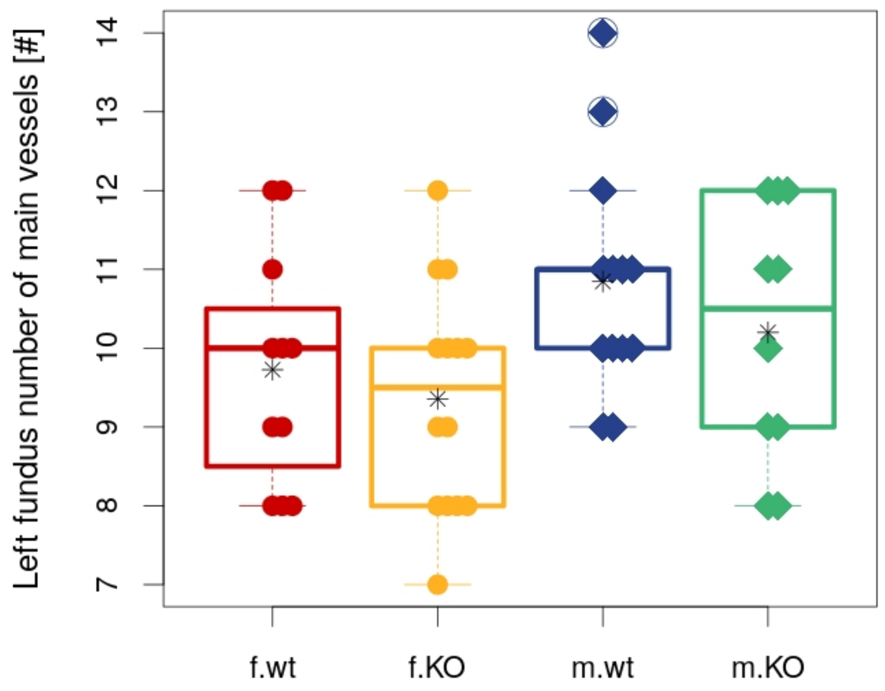

Fundus and retinal main blood vessels are similar to the ones of the control littermates.

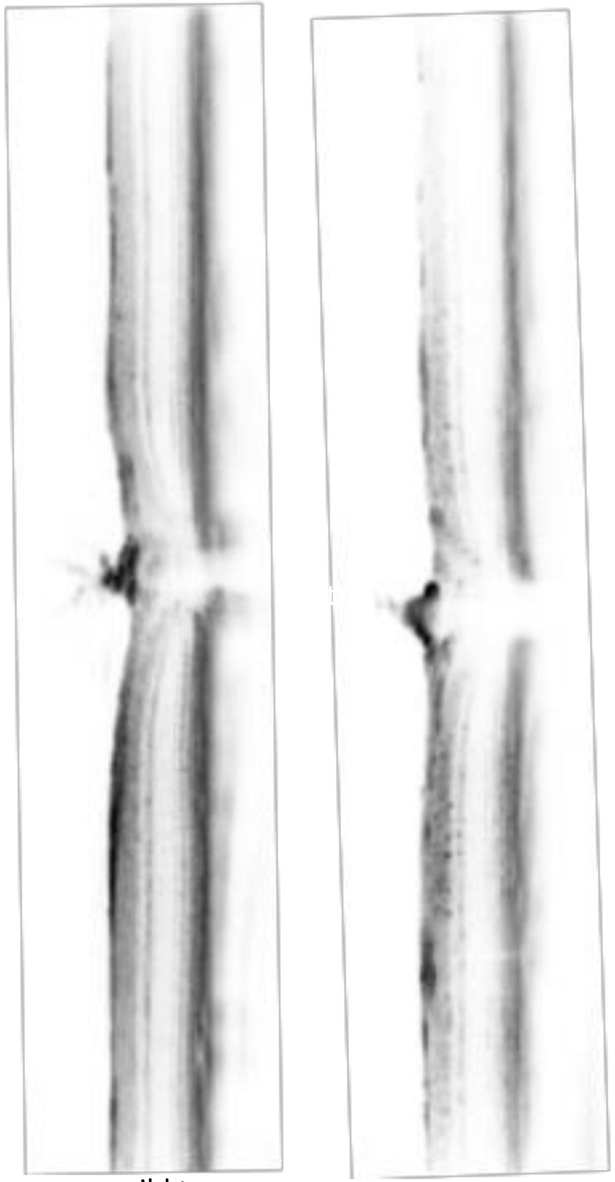

wild type

U90926 KO

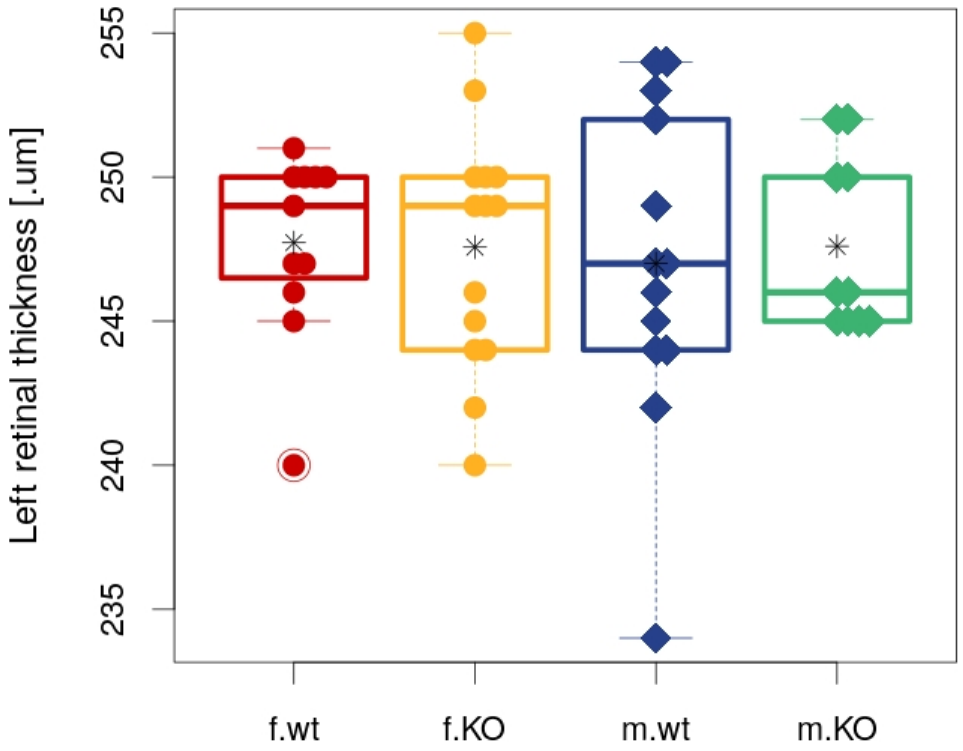

Retinal layers are developed properly and the total retinal thickness is similar to the control littermate retina.

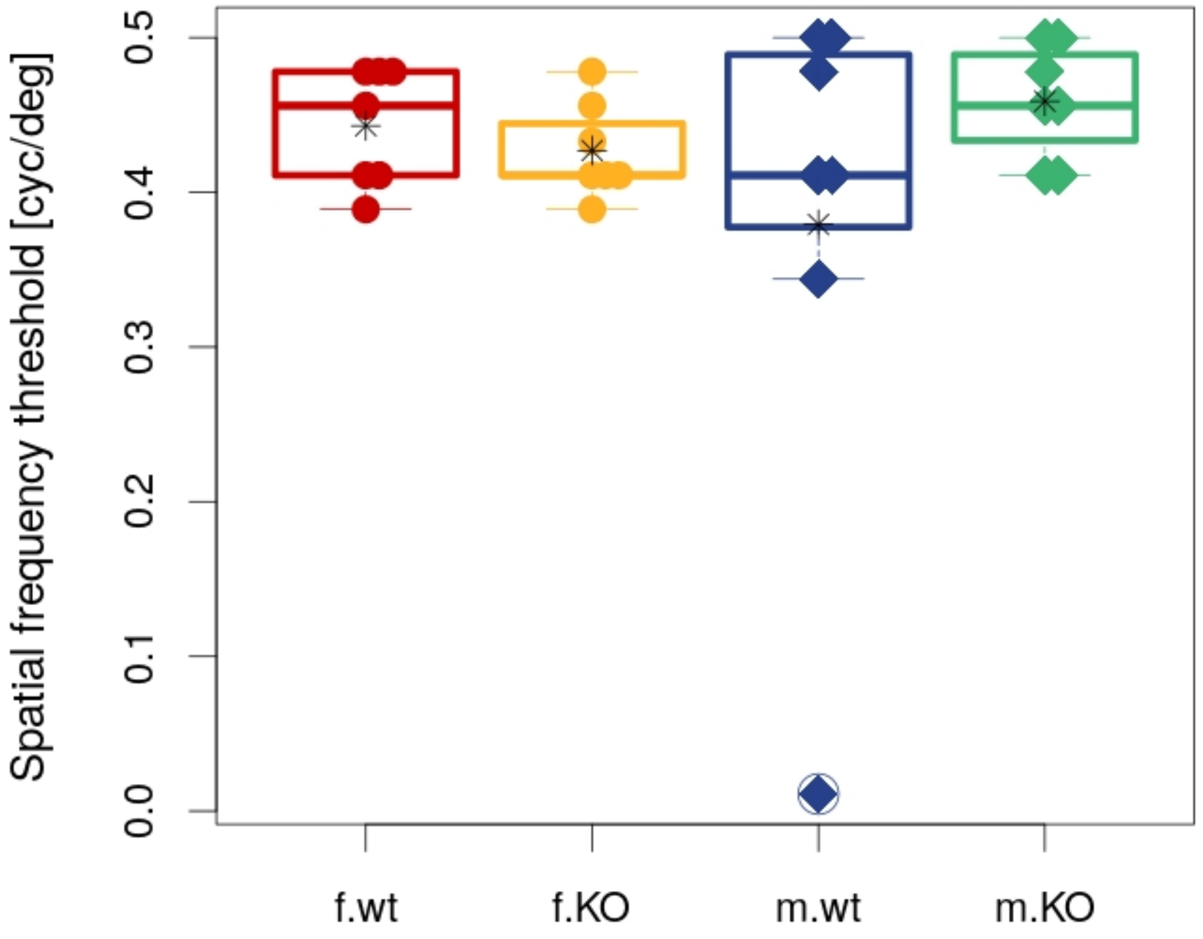

No alteration of the visual function was detected for the *U90926 KO* mutant mouse.

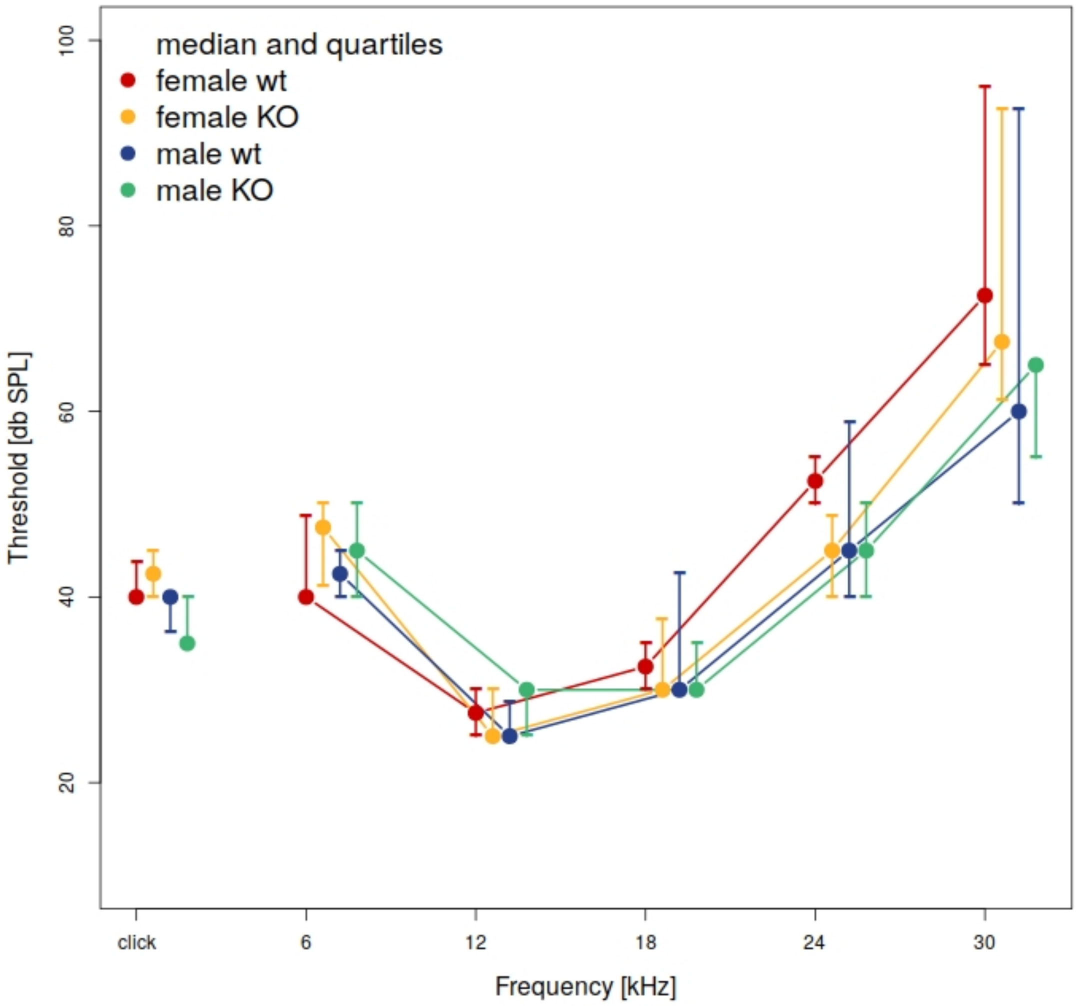

No differences for ABR thresholds

wt

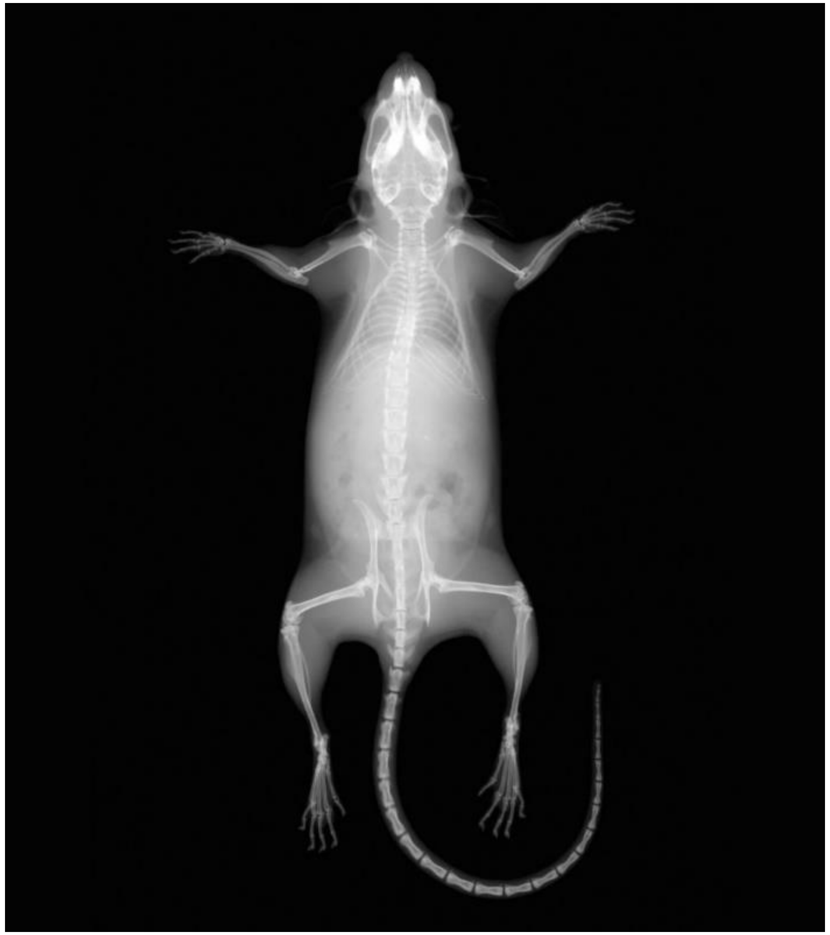

U90926 KO

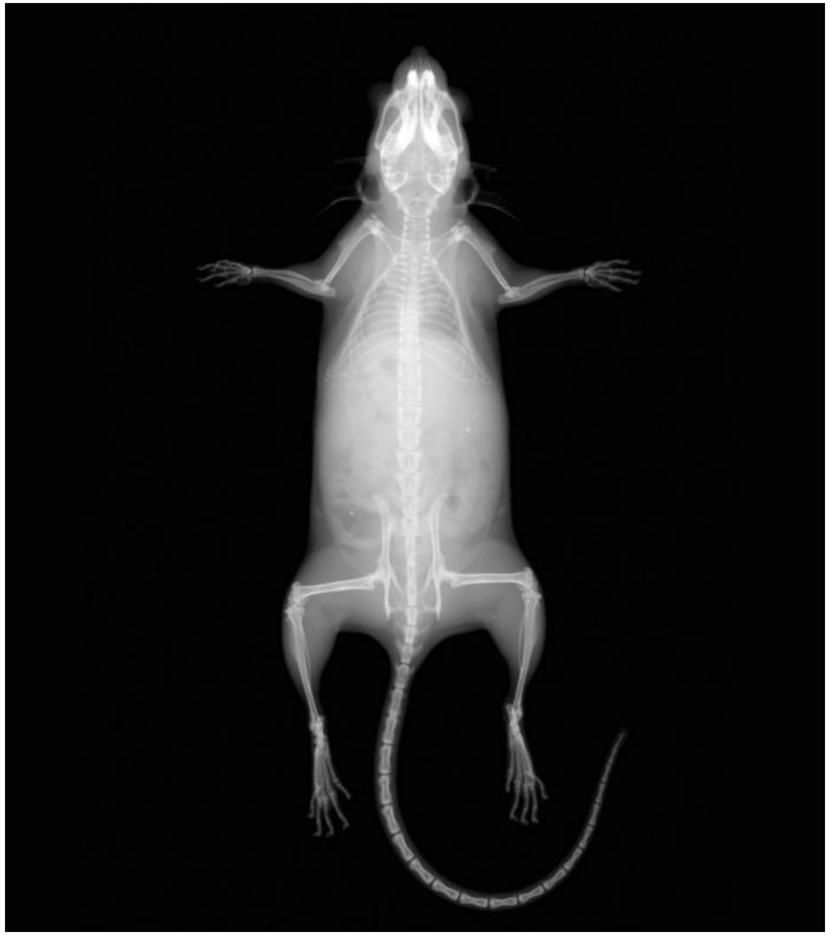

After X-ray analyses, no anatomical alterations were detected in U90926 KO mice

U90926 line: Dual-Energy X-Ray Absorptiometry (DEXA) (17 weeks)

	mean ± sd	mean ± sd	Δ %	ANOVA
	female wt	female KO	KO-wt/wt	genotype
	N=11	N=14		p-value
**Body weight [g]**	23.9 ± 0.9	23.4 ± 1.3	-2.1	0.307
**Total weight whole mouse DXA [g]**	23.4 ± 1.0	23.0 ± 1.3	-1.7	0.379
**Bone area whole mouse [cm²]**	8.17 ± 0.25	7.88 ± 0.24	-3.5	0.008
**BMC whole mouse [mg]**	527.481 ± 23.354	501.166 ± 28.784	-5	0.022
**BMD whole mouse [mg/cm²]**	64.5 ± 1.7	63.5 ± 2.4	-1.6	0.249
**vBMD whole mouse [mg/cm³]**	22.6 ± 0.6	22.6 ± 0.8	0	0.807
**Bone area ROI [cm²]**	0.49 ± 0.03	0.47 ± 0.02	-4.1	0.01
**BMC ROI [mg]**	35.368 ± 3.075	32.571 ± 3.174	-7.9	0.037
**BMD ROI [mg/cm²]**	72.0 ± 4.2	70.0 ± 5.6	-2.8	0.342
**vBMD ROI [mg/cm³]**	92.2 ± 2.8	93.3 ± 3.5	1.2	0.418

	mean ± sd	mean ± sd	Δ %	ANOVA
	male wt	male KO	KO-wt/wt	genotype
	N=13	N=9		p-value
**Body weight [g]**	31.4 ± 1.9	30.7 ± 2.2	-2.2	0.431
**Total weight whole mouse DXA [g]**	31.2 ± 1.9	30.5 ± 2.3	-2.2	0.437
**Bone area whole mouse [cm²]**	8.68 ± 0.27	8.48 ± 0.46	-2.3	0.219
**BMC whole mouse [mg]**	580.105 ± 29.791	576.303 ± 47.167	-0.7	0.819
**BMD whole mouse [mg/cm²]**	66.8 ± 2.1	67.9 ± 2.4	1.6	0.287
**vBMD whole mouse [mg/cm³]**	22.7 ± 0.7	23.3 ± 0.5	2.6	0.031
**Bone area ROI [cm²]**	0.51 ± 0.03	0.53 ± 0.05	3.9	0.39
**BMC ROI [mg]**	35.991 ± 4.098	37.478 ± 5.530	4.1	0.476
**BMD ROI [mg/cm²]**	70.0 ± 5.0	71.0 ± 6.8	1.4	0.688
**vBMD ROI [mg/cm³]**	93.3 ± 3.2	93.7 ± 3.5	0.4	0.795

Decreased bone mineral content (BMC) in female mutants and slightly increased volumetric bone mineral density (vBMD) in male KO mice were detected by DEXA

Body composition analysis II (18 weeks old)

	female		male		linear model	linear model	linear model	linear model
	wt	KO	wt	KO	sex	genotype	Bodymass	sex:genotype
	n=11	n=14	n=14	n=10				
	mean ± sd	mean ± sd	mean ± sd	mean ± sd	p-value	p-value	p-value	p-value
**Body mass NMR**	24 ± 1	22.8 ± 1.3	31.5 ± 1.9	30.1 ± 2	< 0.001	0.007	NA	0.82
**Fat mass NMR**	5.7 ± 1	5.1 ± 0.7	7.5 ± 1.2	6.6 ± 0.9	0.002	0.415	< 0.001	0.484
**Lean mass NMR**	14.8 ± 0.6	14.3 ± 0.5	19.7 ± 1	19.4 ± 1.3	< 0.001	0.356	< 0.001	0.392
**Free fluid**	0.9 ± 0.1	0.9 ± 0.1	1.1 ± 0.2	0.9 ± 0.1	< 0.001	0.621	< 0.001	0.032
**Adiposity Index**	0.4 ± 0.1	0.4 ± 0	0.4 ± 0.1	0.3 ± 0	0.001	0.419	< 0.001	0.658

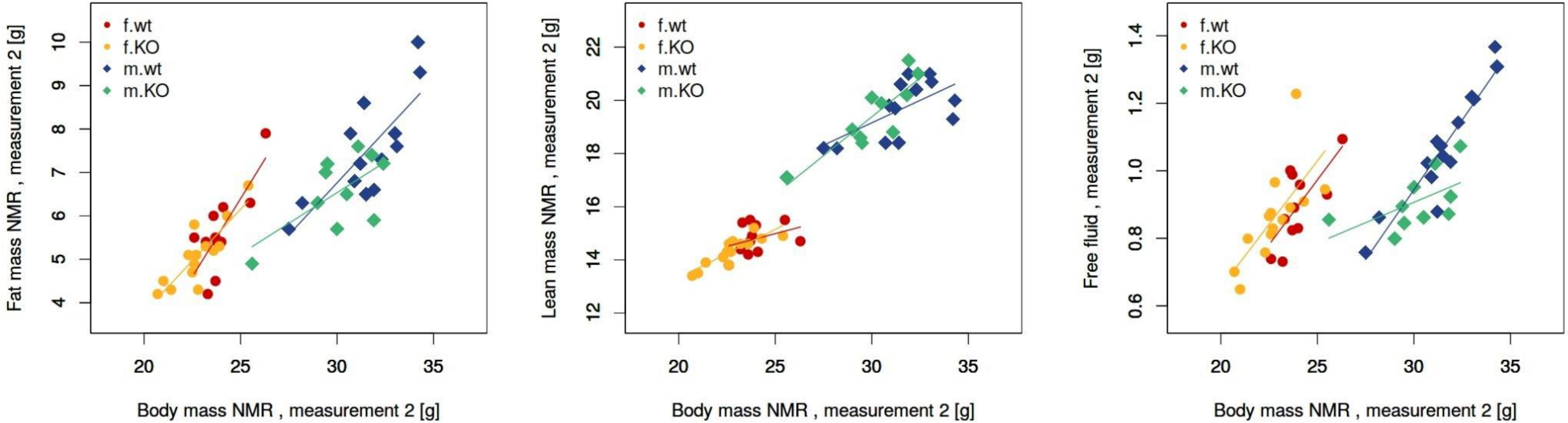

Body composition analysis II (18 weeks old)

	female		male		linear model	linear model	linear model	linear model
	wt	KO	wt	KO	sex	genotype	Bodymass	sex:genotype
	n=11	n=14	n=14	n=10				
	mean ± sd	mean ± sd	mean ± sd	mean ± sd	p-value	p-value	p-value	p-value
**Body mass NMR**	24 ± 1	22.8 ± 1.3	31.5 ± 1.9	30.1 ± 2	< 0.001	0.007	NA	0.82
**Fat mass NMR**	5.7 ± 1	5.1 ± 0.7	7.5 ± 1.2	6.6 ± 0.9	0.002	0.415	< 0.001	0.484
**Lean mass NMR**	14.8 ± 0.6	14.3 ± 0.5	19.7 ± 1	19.4 ± 1.3	< 0.001	0.356	< 0.001	0.392
**Free fluid**	0.9 ± 0.1	0.9 ± 0.1	1.1 ± 0.2	0.9 ± 0.1	< 0.001	0.621	< 0.001	0.032
**Adiposity Index**	0.4 ± 0.1	0.4 ± 0	0.4 ± 0.1	0.3 ± 0	0.001	0.419	< 0.001	0.658

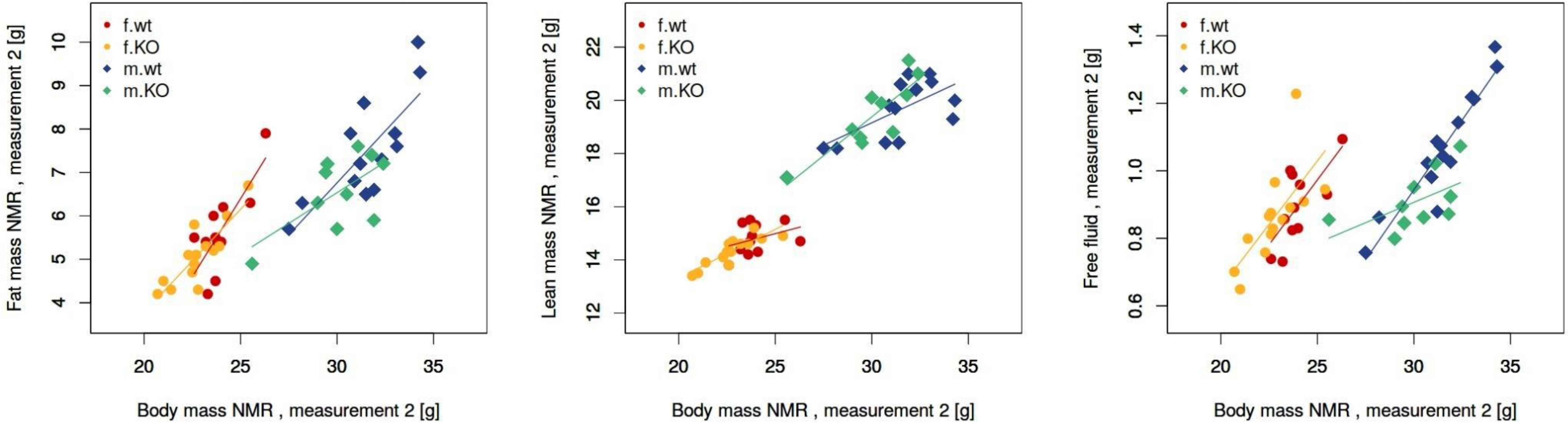

Mutants showed a subtle genotype-dependent body weight reduction

Body composition analysis (13-18 weeks old)

	sex	genotype	measurement	Bodymass	genotype:measurement
**Body mass NMR**	< 0.001	0.027	< 0.001	NA	< 0.001
**Fat mass NMR**	< 0.001	0.905	0.438	< 0.001	0.044
**Lean mass NMR**	< 0.001	0.744	0.923	< 0.001	0.026

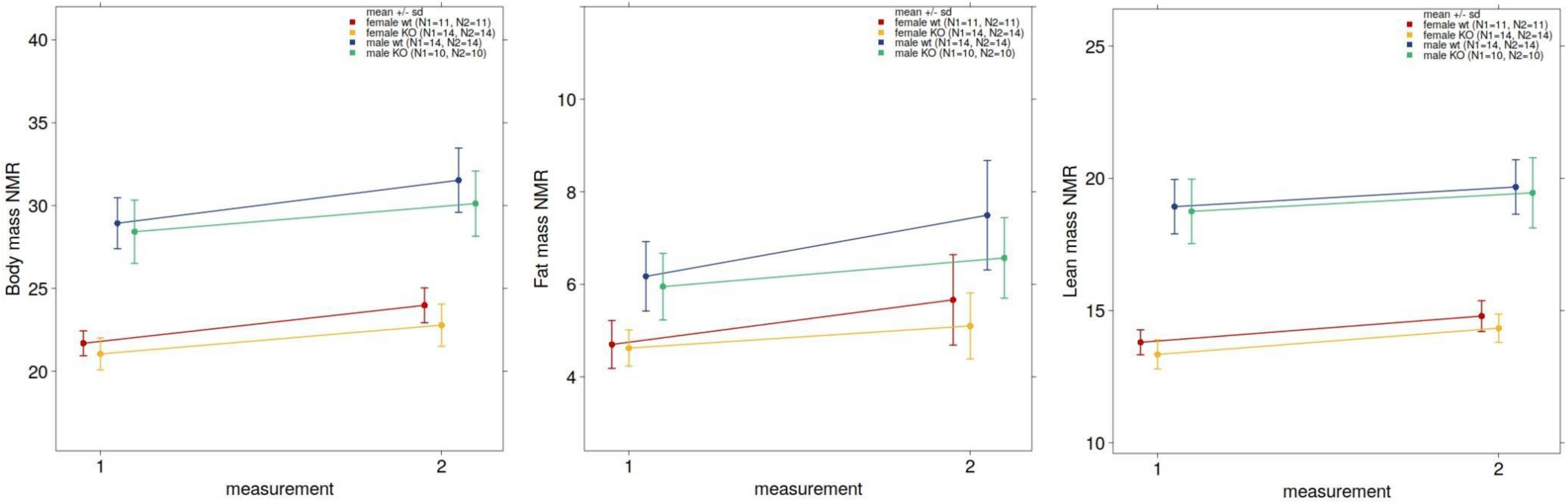

Tendency in gaining less fat amount with ageing

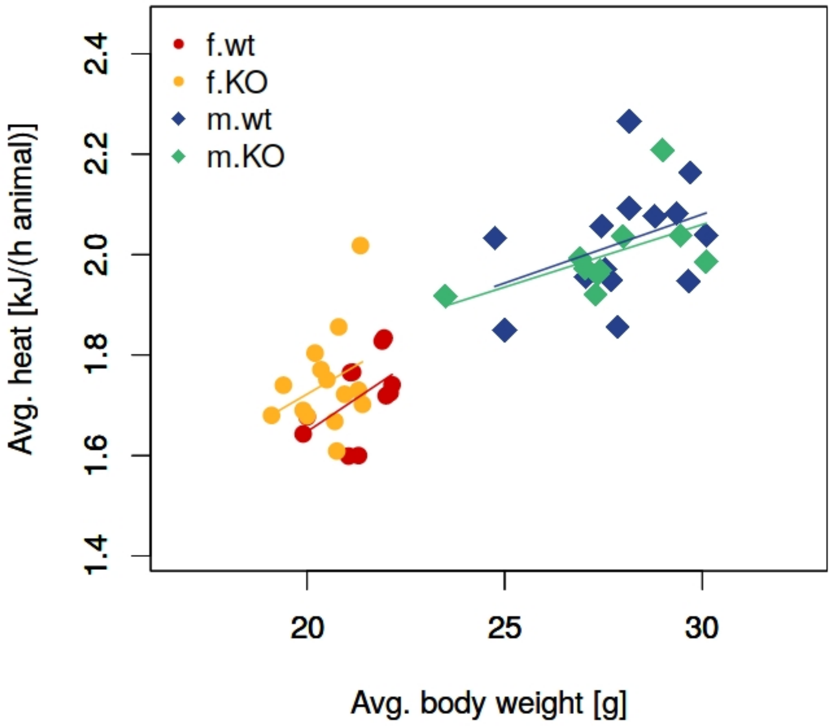

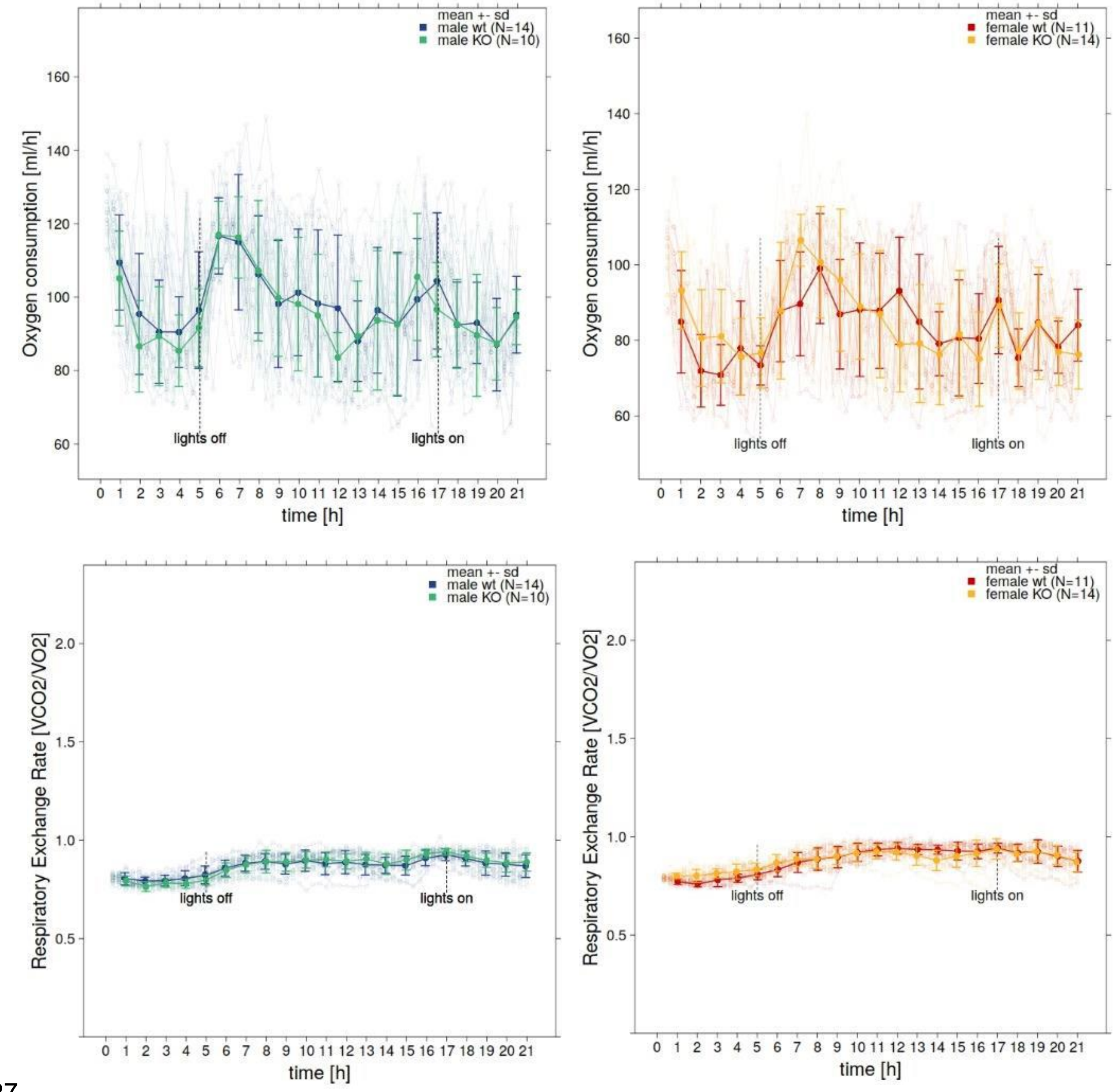

Slightly higer metabolic rate in females

Matching max oxygen consumption rates

U90926_KO	female		male		linear model	linear model	linear model
	wt	KO	wt	KO	genotype	sex	genotype:sex
	n=11	n=14	n=14	n=10			
	mean ± sd	mean ± sd	mean ± sd	mean ± sd	p-value	p-value	p-value
**RBC [Mio/mm³]**	10.45 ± 0.41	10.4 ± 0.53	10.17 ± 0.36	10.12 ± 0.69	0.738	0.059	0.97
**HGB [g/dl]**	16.38 ± 0.78	16.25 ± 0.84	15.56 ± 0.52	15.52 ± 1.05	0.704	0.002	0.85
**HCT [%]**	50.29 ± 2.01	49.36 ± 2.5	47.79 ± 1.5	47.7 ± 2.89	0.438	0.002	0.519
**MCV [fl]**	48.18 ± 0.98	47.57 ± 1.55	47.14 ± 1.1	47.1 ± 0.57	0.331	0.028	0.398
**MCH [pg]**	15.67 ± 0.55	15.62 ± 0.47	15.31 ± 0.42	15.36 ± 0.63	0.996	0.04	0.726
**MCHC [g/dl]**	32.57 ± 0.79	32.91 ± 0.59	32.59 ± 0.61	32.54 ± 1.21	0.526	0.439	0.407
**RDW [%]**	13.32 ± 0.32	14.24 ± 3.73	14.47 ± 0.98	14.75 ± 0.57	0.329	0.175	0.6
**WBC [10³/mm³]**	4.99 ± 1.86	4.94 ± 1.29	6.87 ± 2.12	7.11 ± 1.5	0.855	< 0.001	0.77
**PLT [10³/mm³]**	973.36 ± 130.33	981.43 ± 188.33	1234.86 ± 115.8	1232.9 ± 145.08	0.944	< 0.001	0.908
**MPV [fl]**	6.55 ± 0.25	6.51 ± 0.17	6.29 ± 0.13	6.27 ± 0.16	0.561	< 0.001	0.883
**PDW [fL]**	6.03 ± 0.41	5.99 ± 0.3	5.61 ± 0.22	5.59 ± 0.25	0.738	< 0.001	0.889
**PLCR [%]**	2.97 ± 0.72	2.69 ± 0.57	2.05 ± 0.36	2.09 ± 0.46	0.431	< 0.001	0.298
**PCT [%]**	0.64 ± 0.08	0.64 ± 0.13	0.78 ± 0.08	0.77 ± 0.08	0.914	< 0.001	0.863

No evidence of genotype-related effects on hematological parameters.

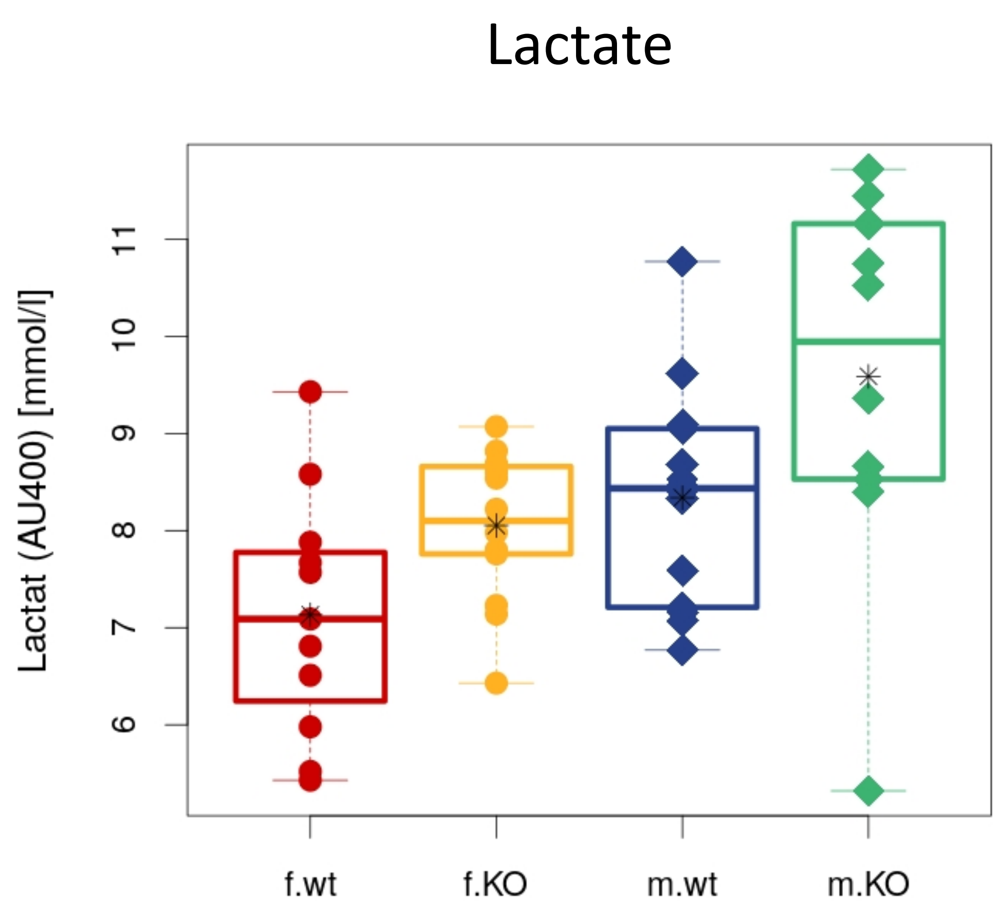

Slightly elevated lactate levels in mutant animals as compared to controls.
Linear model, genotype effect p = 0,005

Results flow cytometry_main leukocyte lineages (19 weeks)

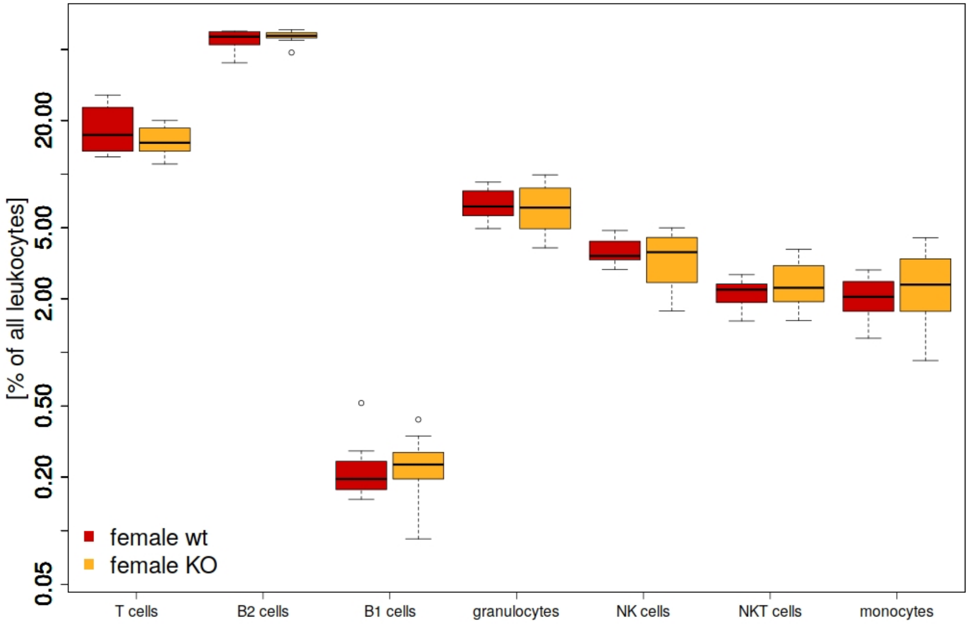

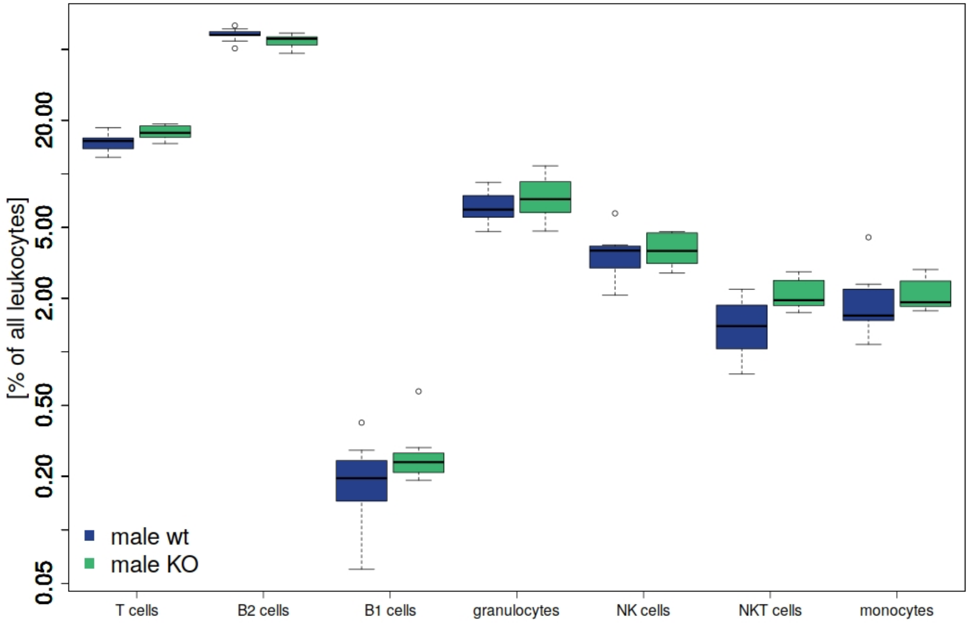

	female		male		female	male	overall
	wt	KO	wt	KO			
	n=8	n=12	n=12	n=7			
	median [25%, 75%]	median [25%, 75%]	median [25%, 75%]	median [25%, 75%]	p-value	p-value	p-value
**T cells**	16.6 [13.88 , 23.2]	15 [13.97 , 17.62]	15.3 [14.03 , 15.8]	17 [16.05 , 18.6]	0.353	0.017	0.545
**B2 cells**	59.1 [54.27 , 62.95]	59.75 [58.33 , 61.9]	60.3 [59.95 , 62.45]	57.4 [52.95 , 58.8]	0.72	0.027	0.222
**B1 cells**	0.2 [0.18 , 0.23]	0.24 [0.2 , 0.27]	0.2 [0.15 , 0.23]	0.24 [0.21 , 0.27]	0.314	0.104	0.047
**granulocytes**	6.58 [6.06 , 7.7]	6.49 [4.97 , 8.32]	6.3 [5.76 , 7.34]	7.21 [6.06 , 9.04]	0.571	0.34	0.829
**NK cells**	3.48 [3.37 , 4.19]	3.65 [2.51 , 4.27]	3.7 [3.08 , 3.92]	3.69 [3.13 , 4.66]	0.851	0.522	0.873
**NKT cells**	2.25 [1.98 , 2.42]	2.31 [1.96 , 3.03]	1.4 [1.04 , 1.82]	1.95 [1.82 , 2.52]	0.508	0.014	0.007
**monocytes**	2 [1.7 , 2.4]	2.4 [1.8 , 3.3]	1.6 [1.5 , 2.2]	1.9 [1.8 , 2.5]	0.415	0.112	0.101

	female		male		female	male	overall
	wt	KO	wt	KO			
	n=8	n=12	n=12	n=7			
	median [25%, 75%]	median [25%, 75%]	median [25%, 75%]	median [25%, 75%]	p-value	p-value	p-value
**T/CD4+**	55.1 [52.92 , 56.3]	54.05 [52.3 , 57.67]	54.7 [52.9 , 55.88]	54.5 [52.55 , 54.9]	0.851	0.837	0.776
**T/CD8+**	41.5 [39.55 , 43.83]	41.25 [38.85 , 42.83]	42.4 [41.02 , 43.3]	42.5 [41.6 , 44.55]	0.836	0.664	0.873
**T/DP**	0.2 [0.18 , 0.27]	0.32 [0.22 , 0.38]	0.29 [0.26 , 0.37]	0.43 [0.31 , 0.46]	0.314	0.047	0.177
**T/DN**	3.67 [2.84 , 3.9]	4.13 [3.54 , 5.09]	3.06 [2.55 , 3.59]	2.51 [2.34 , 2.94]	0.215	0.261	0.518
**T/gdTCR+**	2.25 [2.03 , 2.75]	2.96 [2.6 , 3.42]	2.04 [1.94 , 2.28]	1.96 [1.83 , 2.1]	0.175	0.419	0.448
**gdTCR+/DN**	84.7 [81.38 , 89.83]	89.7 [88.4 , 92.58]	87.25 [84.8 , 91.25]	88.9 [84.75 , 91.45]	0.129	0.853	0.177

	female		male		female	male	overall
	wt	KO	wt	KO			
	n=8	n=12	n=12	n=7			
	median [25%, 75%]	median [25%, 75%]	median [25%, 75%]	median [25%, 75%]	p-value	p-value	p-value
**Ly6C low monocytes**	26.6 [23.1 , 30.3]	26.1 [21.4 , 37]	22.4 [16 , 30.5]	18.5 [13.3 , 19.9]	0.91	0.261	0.923
**Ly6C intermediate monocytes**	16.6 [13.6 , 19.4]	19.6 [15.3 , 21.9]	15.9 [12.5 , 18.3]	16.9 [11.9 , 17.9]	0.262	0.983	0.261
**Ly6C high monocytes**	36 [23.2 , 40.8]	33.2 [20.6 , 46]	38.8 [31 , 53.4]	44.6 [40.8 , 48]	0.97	0.711	0.972

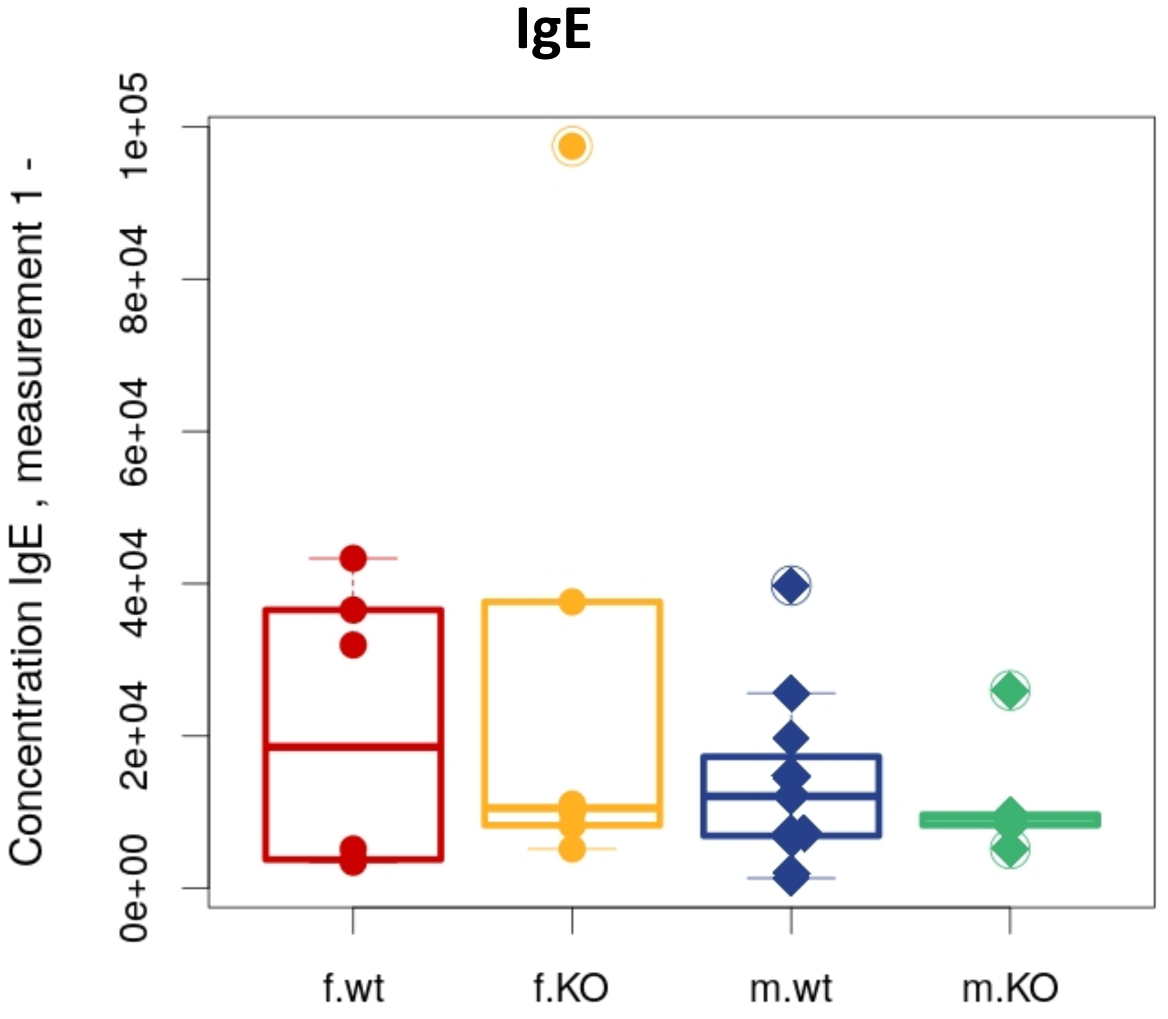

No phenotype

Means, Standard deviations and p-values of a Wilcoxon test.

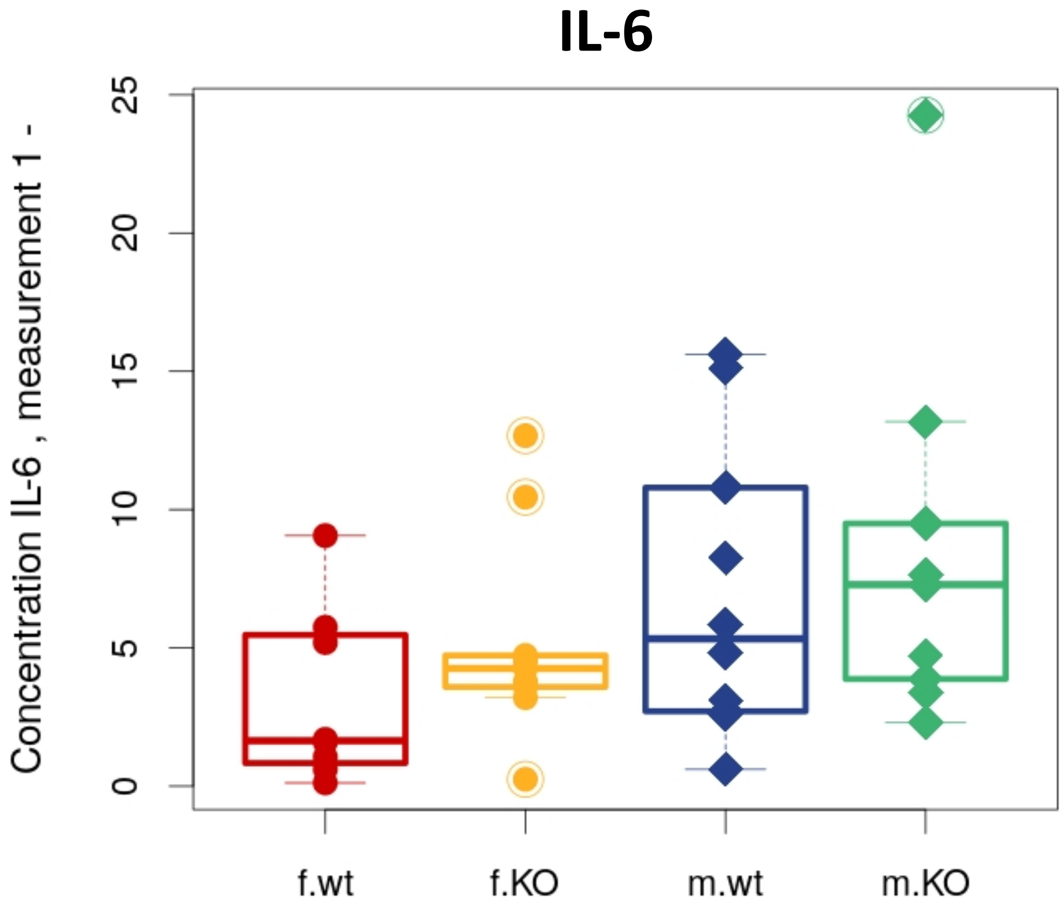

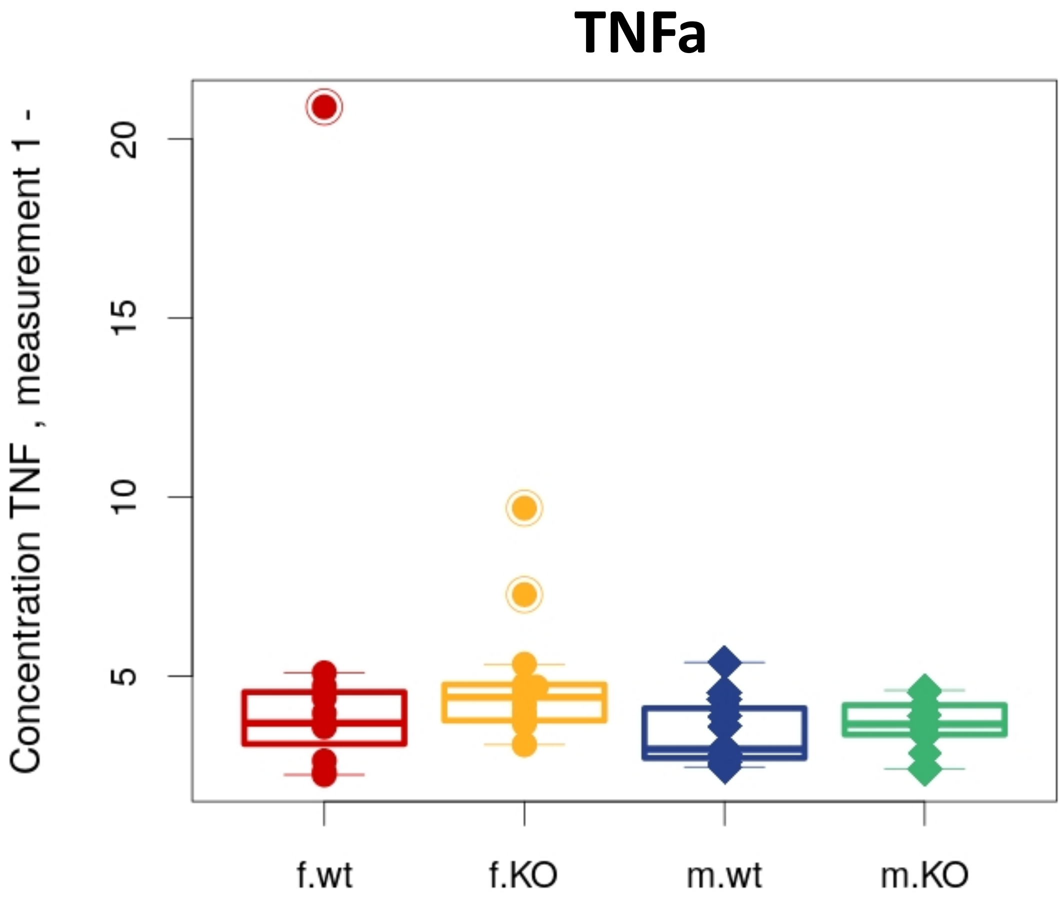

Means, Standard deviations and p-values of a Wilcoxon test.

Means, standard deviation and p-values calculated by a linear model (parameter ~ genotype * sex + body weight)

	female		male		linear model	linear model	linear model	linear model
	wt	KO	wt	KO	genotype	sex	bodyWeight	genotype:sex
	n=5	n=5	n=5	n=5				
	mean ± sd	mean ± sd	mean ± sd	mean ± sd	p-value	p-value	p-value	p-value
**Heart weight [mg]**	134 ± 19.1	137.2 ± 10.2	164.8 ± 20.4	184.4 ± 28.7	0.499	0.578	0.081	0.803
**Tibia length [mm]**	18.22 ± 0.47	18.19 ± 0.48	18.6 ± 0.18	18.78 ± 0.47	0.732	0.27	0.924	0.635
**Age of mice when culled [day]**	141 ± 2	141 ± 2	143 ± 2	140 ± 1	0.08	0.739	0.951	0.18
**Liver weight [g]**	1.291 ± 0.233	1.226 ± 0.191	1.694 ± 0.109	1.705 ± 0.115	0.652	0.031	0.679	0.761
**Spleen weight [g]**	0.099 ± 0.025	0.086 ± 0.007	0.079 ± 0.009	0.088 ± 0.018	0.796	0.429	0.81	0.232

Histological findings – genotype-specific differences (19 weeks)

Skin	NAD	Oesophagus	NAD
Brain	NAD	Stomach	NAD
Cervical lymph node	NAD	Small intestine	NAD
Salivary glands	NAD	Large intestine	NAD
Trachea	NAD	Pancreas	NAD
Thyroid	NAD	Testes	NAD
Parathyroid	NAD	Epididymides	NAD
Heart	NAD	Funiculus spermaticus	NAD
Lung	NAD	Prostate	NAD
Thymus	NAD	Male accessory glands	NAD
Spleen	NAD	Ovaries	NAD
liver	NAD	Uterus	NAD
Adrenals	NAD	Vagina	NAD
Kidney	NAD	Urinary bladder	NAD
WAT	NAD	BAT	NAD

NAD: no alterations determined
