# Supplementary material for: LncRNA U90926 is dispensable for the development of obesity‐associated phenotypes in vivo
Source: Physiol Rep. 2024 Jan 3;12(1):e15901. doi: 10.14814/phy2.15901 (PMC10764201; doi:10.14814/phy2.15901)
Supplement: Supplementary file 2 — Figure S2. [file PHY2-12-e15901-s001.docx]

**Figure S2: *U90926* expression is downregulated in 3T3-L1 cells in long-term cell culture regardless of differentiation.** 3T3-L1 cells were plated for an eight-day differentiation protocol with or without differentiation mix (1μM dexamethasone + 0.5mM IBMX + 1μg/ml insulin). Samples were collected every 2 days, followed by RT-qPCR to measure *U90926* expression. All the data are expressed relative to the housekeeping gene, *B2m* and calculated by a comparative Ct method formula 2^-(deltaCt) and multiplied by a factor of 10,000 for ease of visualization. Data are represented as the mean ± SEM.
