# Supplementary material for: LncRNA U90926 is dispensable for the development of obesity‐associated phenotypes in vivo
Source: Physiol Rep. 2024 Jan 3;12(1):e15901. doi: 10.14814/phy2.15901 (PMC10764201; doi:10.14814/phy2.15901)
Supplement: Supplementary file 3 — Figure S3. [file PHY2-12-e15901-s003.docx]

**Fig. S3 HFD-fed WT mice demonstrate higher weight gain compared with ND-fed WT mice.** WT C57BL/6J (B6) mice were analyzed in two groups, normal diet (ND)-fed (n=7) and high-fat diet (HFD)-fed (n=6). HFD was introduced at the age of 8 weeks and continued for another 8 weeks. Weights were taken every 4 weeks until 16 weeks of age for both groups. Weight gain comparison in ND-fed and HFD-fed mice are shown here in males and females (panel A and B). Data are represented as the mean ± SEM. For statistical analysis, two-way ANOVA was performed, and Šídák's multiple comparisons test was performed as a post-hoc analysis. Here, P values are shown as *= 0.02, **=0.002, and ***=<0.001.
